# Global prevalence of physical activity for children and adolescents; inconsistencies, research gaps, and recommendations: a narrative review

**DOI:** 10.1186/s12966-021-01155-2

**Published:** 2021-06-29

**Authors:** Salomé Aubert, Javier Brazo-Sayavera, Silvia A. González, Ian Janssen, Taru Manyanga, Adewale L. Oyeyemi, Patrick Picard, Lauren B. Sherar, Evan Turner, Mark S. Tremblay

**Affiliations:** 1 Healthy Active Living and Obesity Research Group, CHEO Research Institute, Ottawa, Ontario Canada; 2 PDU EFISAL, Centro Universitario Regional Noreste, Universidad de la República, Rivera, Uruguay; 3 Department of Sports and Computer Science, Universidad Pablo de Olavide (UPO), ES-41013 Seville, Spain; 4 School of Kinesiology and Health Studies, and Department of Public Health Sciences, Queen’s University, Kingston, Ontario Canada; 5 School of Epidemiology and Public Health, University of Ottawa, Ottawa, Ontario Canada; 6 Department of Physiotherapy, College of Medical Sciences, University of Maiduguri, Maiduguri, Nigeria; 7 School of Sport, Exercise and Health Sciences, Loughborough University, Loughborough, UK; 8 Centre for Global Child Health and SickKids Research Institute, The Hospital for Sick Children, Toronto, Ontario Canada

**Keywords:** Global surveillance, Physical activity questionnaire, International comparisons, Accelerometry, Youth

## Abstract

**Background:**

One of the strategic actions identified in the Global Action Plan on Physical Activity (PA) 2018–2030 is the enhancement of data systems and capabilities at national levels to support regular population surveillance of PA. Although national and international standardized surveillance of PA among children and adolescents has increased in recent years, challenges for the global surveillance of PA persist. The aims of this paper were to: (i) review, compare, and discuss the methodological inconsistencies in children and adolescents’ physical activity prevalence estimates from intercontinental physical activity surveillance initiatives; (ii) identify methodological limitations, surveillance and research gaps.

**Methods:**

Intercontinental physical activity surveillance initiatives for children and adolescents were identified by experts and through non-systematic literature searches. Prevalence of meeting PA guidelines by country, gender, and age were extracted when available. A tool was created to assess the quality of the included initiatives. Methods and PA prevalence were compared across data/studies and against the methodological/validity/translation differences.

**Results:**

Eight intercontinental initiatives were identified as meeting the selection criteria. Methods and PA definition inconsistencies across and within included initiatives were observed, resulting in different estimated national prevalence of PA, and initiatives contradicting each other’s cross-country comparisons. Three findings were consistent across all eight initiatives: insufficient level of PA of children and adolescents across the world; lower levels of PA among girls; and attenuation of PA levels with age. Resource-limited countries, younger children, children and adolescents not attending school, with disability or chronic conditions, and from rural areas were generally under/not represented.

**Conclusions:**

There are substantial inconsistencies across/within included initiatives, resulting in varying estimates of the PA situation of children and adolescents at the global, regional and national levels. The development of a new PA measurement instrument that would be globally accepted and harmonized is a global health priority to help improve the accuracy and reliability of global surveillance.

**Supplementary Information:**

The online version contains supplementary material available at 10.1186/s12966-021-01155-2.

## Background

The benefits of physical activity (PA) for the health and well-being of children and adolescents are now widely accepted by the international scientific community [1, 2]. Specific PA recommendations for the early years (0–4 years) and for children and adolescents (5–17 years) to improve fitness and health have been published by numerous countries [3] and the World Health Organization (WHO) [4–6]. Aligned with the United Nations’ *Sustainable Development Goals* (SDGs), [7] the WHO published the *Global Action Plan on Physical Activity 2018–2030* to provide guidance to support the implementation of national multi-sectoral PA actions and set a specific target of a 15% relative reduction in the global prevalence of physical inactivity in adults and adolescents [8]. One of the strategic actions identified in the Global Action Plan is the enhancement of data systems and capabilities at national levels to support regular population surveillance of PA.

Over the past 20 years, several questionnaires and surveys [9–15] have been developed in part to estimate the PA levels of children and adolescents. Several of these questionnaires have been used in large scale international studies to estimate population levels of PA in a standardized way and to allow for comparisons across countries, regions, and studies [12, 13, 15–18]. Other types of international initiatives, including syntheses of all available evidence on the levels of PA of children and adolescents within and between nations, [19, 20] and the harmonization of accelerometer data with the aim of obtaining standardized PA prevalence estimates, [21, 22] were established more recently.

Although national and international standardized surveillance of PA among children and adolescents has increased in recent years, challenges for the global surveillance of PA and lack of generalization of PA data between and across continents persist. For example, there are methodological inconsistencies across international PA surveillance initiatives and national PA prevalence estimates often differ from one surveillance initiative to the next [23–26]. Moreover, the extent of the methodological inconsistencies across international PA surveillance initiatives between and across different continents have rarely been empirically documented.

The aim of this paper is to review, compare, and discuss the methodological inconsistencies in children and adolescents’ PA prevalence estimates from available intercontinental PA surveillance data/studies. This paper also aims to identify methodological limitations, surveillance and research gaps, and propose recommendations for improvement.

## Methods

To control for over-representation of physical activity surveillance initiatives limited to high-income country settings and to centre the discussion around the physical activity data most commonly referred to in major publications and generally used to inform public health institutions, intercontinental PA surveillance initiatives were identified by international experts and literature searches. The inclusion criteria for the initiatives were the following:

- collected or compiled data from the year 2000 onwards;
- included multiple countries from at least two continents;
- focused on early years (0–4 years) and/or children and adolescents (5–17 years);
- early years, children, and adolescents of all ability/needs levels and health status;
- assessed/estimated PA using report and/or device measures (e.g. accelerometers, pedometers);
- dataset presents publicly available estimated prevalence of PA at national levels.

Prevalence estimates of children and/or adolescents meeting moderate- to vigorous-intensity PA guidelines were extracted by country, gender, and age (or school grade) when available. The 2016 physical *inactivity* estimates from Guthold et al. (2020) [20] were extracted, then converted into prevalence of PA. PA prevalence from the Global School Health Survey (GSHS) was extracted in priority from the WHO Country Fact Sheets when available, then from the available GSHS national reports. GSHS regional prevalences were extracted when national prevalences were not available. PA prevalence regional averages were calculated using a simple average calculation, except for the data from Guthold et al. (2020) where the already available PA pooled regional averages were directly extracted. Methods for each included PA surveillance initiative (e.g., sample size, age or grade of participants, year(s) of data collection) were extracted. The following information was extracted from initiatives using questionnaires to assess PA: adherence to PA guidelines, items assessing PA, languages available for the questionnaires, questionnaire validity, and the method used for their translation and cultural adaptation. For initiatives using device-based measurements, the definition for adherence to PA guidelines, type of device, minimum wear time (valid daily wear time and criteria for a valid file), and the intensity cut-off used for the analysis were extracted.

A tool was created to assess the quality of the included initiatives. A list of relevant criteria for the international surveillance of PA of children and adolescents was identified and agreed upon by the authors after reviewing existing quality assessment tools and a final tool created where a score of 0 = not met, 1 = partially met, and 2 = fully met. Two independent reviewers (SA and ET) used this tool to assign a score to each included initiative and a third reviewer (MST) resolved conflicts between the scores. Equal weighting was given in calculating the average scores.

Finally, tables were created to contrast the methods and results across the included intercontinental PA datasets and to map PA prevalence against the observed methods/validity/translation differences.

## Results

Eight intercontinental initiatives were identified as meeting the selection criteria. Their detailed description and characteristics are presented in Online Supplement Material 1. Two of the initiatives were large international surveys where the PA level of adolescents was assessed by questionnaire (Health Behaviour in School-aged Children [HBSC] study and GSHS) [11, 27]; one involved the standardized measurement of PA of the 9–11 year-olds using accelerometers in 13 countries (International Study of Childhood Obesity, Lifestyle and the Environment [ISCOLE]) [21]; three were from publications presenting statistical analyses of international compilations of available PA datasets for adolescents from multiple sources [20, 28, 29]; one involved the compilation of the best available PA evidence for 5–17 year-olds within 49 countries (Global Matrix 3.0) [30]; and one included the pooling and standardized reduction of accelerometry datasets for 3–18 year-olds (International Children’s Accelerometry Database [ICAD] 1.0) [22].

Online Supplement Material 2 presents the extracted national PA prevalence for children and adolescents by initiative, presented by country and by gender and age (or school grade) when available. The Guthold et al. study was the initiative with the greatest number of national PA prevalences presented, with data available for 146 countries, territories, and areas across six continents,^15^ followed by the Marques et al. study (106 countries or territories across six continents), [29] the GSHS survey (87 countries or territories across six continents), [11] Xu et al. study (54 countries or territories across four continents), [28] the Global Matrix 3.0 (49 countries or territories across six continents), [30] 2017/2018 HBSC study (48 countries or territories across three continents), [31] ISCOLE study (13 countries across six continents), [21, 32] and ICAD 1.0 (10 countries across four continents) [22]. Depending on the initiative, the countries with the highest estimated PA were Bangladesh, [20, 28, 29, 33] Slovenia, [30] Finland, [31] Mozambique, [32] and Norway [34]; while the countries with the lowest estimated PA were South Korea, [20] Belgium, China, Taiwan, Scotland and UAE, [30] Italy, [31] Philippines, [11] China, [35] United States of America (USA), [34] and Cambodia [28, 29]. A detailed summary of the characteristics of the datasets by world region for each included initiative is in Online Supplement Material 3.

Online Supplement Material 4 presents the extraction of information regarding the questionnaires used in the GSHS and the HBSC surveys, including PA questionnaire items, translation, validity, and reliability information.

For the quality assessment process, the percent agreement between the two independent reviewers was 78%. A total of 35 conflicts out of 160 scores (20 scores for each included initiative) were observed between the two independent reviewers before being resolved by the third reviewer. The final scores from the quality assessment of each included initiative are presented in Table 1.

**Table 1:** Scores from the quality assessment of each included initiatives

Criteria/Initiative	Guthold et al. (2020) [20]	Global Matrix 3.0 (30)	HBSC 2017/ 2018 [31]	GSHS (WHO country Factsheet/ national reports)	ISCOLE study [32, 35]	ICAD 1.0 (34)	Marques et al. (2020) [29]	Xu et al. (2020) [28]	Average score
1. Clear description of the objectives of the initiative	2	2	2	2	2	2	2	2	2.0
2. Clear description of the population of interest (e.g., age group, location, gender, ability/need status)	2	2	2	1	2	2	2	2	1.9
3. Clear description of the time of the data collection/compilation (e.g. date, season)	1	1	2	1	2	2	0	1	1.2
4. Clear description of the sampling frame and recruitment methods	1	0	2	2	2	2	0	2	1.4
5. Clear description of the sample’s key characteristics (e.g., size, age, % of male/female)	1	1	2	1	2	2	2	2	1.6
6. Clear description of methods of data collection/ extraction/ compilation for physical activity	2	2	2	2	2	2	2	2	2.0
7. Clear description of the definition of physically active/inactive used	2	2	2	2	2	2	2	2	2.0
8. Adequate measurement of physical activity (validity, reliability)	1	1	1	1	2	2	1	1	1.2
9. Clear description of the data reduction, data analysis and statistical methods employed	2	2	1	1	2	2	2	2	1.7
10. Appropriate interpretation of the statistical findings	1	2	2	2	2	2	2	2	1.9
11. Adequate harmonization and validity of cross-country comparison	1	1	1	1	2	2	1	1	1.2
12. Presentation of specific PA prevalence by country	2	2	2	2	2	2	2	2	2.0
13. Presentation of specific PA prevalence by gender	2	0	2	2	1	2	1	2	1.5
14. Presentation of specific PA prevalence by socioeconomic status	1	1	2	0	0	0	1	0	0.6
15. Presentation of specific PA prevalence by age groups	1	0	2	2	2	2	1	2	1.5
16. Presentation of specific PA prevalence for children and adolescents with disability or chronic condition	0	0	0	0	0	0	0	0	0
17. Representativeness of both urban and rural populations	2	1	1	2	0	1	1	2	1.2
18. Representativeness of children and adolescents not attending school	0	0	0	0	0	1	1	0	0.2
19. Samples are nationally representative samples	2	1	2	2	0	1	2	2	1.5
20. Appropriate description of who analyzed and reported the data	2	1	2	1	2	2	2	2	1.7
Total	28	22	32	27	29	33	27	31	28.6

**Notes:** *HBSC* Health Behaviour in School-aged Children; *GSHS* Global School-Based Student Health Survey; *ISCOLE* International Study of Childhood Obesity, Lifestyle and the Environment; *ICAD* International Children’s Accelerometry Database. The full extracted datasheet is available from the corresponding author by reasonable request. 0 = criterion not met, 1 = criterion partially met, and 2 = criterion fully met

## Discussion

Eight intercontinental PA surveillance initiatives were identified for children and adolescents with open access to their findings and/or data and ranged from 10 [34] to 146 [20] countries. With the exceptions of the 2017/2018 HBSC study and ICAD 1.0 that focused predominantly on European and high-income western countries, there was wide geographic distribution. It is important to note that the GSHS survey, with data from low-and middle-income countries (LMICs) across all continents and included as a standalone initiative in the present review, was also the main source of data for three other included initiatives, [20, 28, 29] constituting a potential for overrepresentation of the same data. In addition, data from the previous version of the HBSC study (i.e., 2014 HBSC) was also a main source of data for two other included initiatives [20, 29].

### Comparison of the age ranges, timing of data collection, and representativeness

Six of eight initiatives included only children and adolescents aged nine or above in their targeted study population [11, 20, 28, 29, 31, 35]. The ICAD 1.0 included accelerometery data from 3 to 18 year-old children and adolescents, however, estimates of PA levels were only available for 9–10 and 12–13 year-olds [34]. Conversely, the Global Matrix 3.0, with a 5–17 year-old targeted study population, was the only initiative that reported national physical activity levels for children aged 5–9 years old [30]. However, the age ranges for all the datasets included in the Global Matrix 3.0 presented in Online Supplement Material 3 show that adolescents aged ≥10 were more represented than younger children. None of the included initiative presented data for the early years (< 5 years old).

The HBSC study and the Global Matrix were the only surveillance initiatives with an established periodicity. The HBSC has occurred every four years since 1982 [27] and the Global Matrix took place every two years between 2014 and 2018, [30] and the Global Matrix 4.0 [36] is planned to be launched in 2022. Online Supplement Material 3 shows that the datasets used in the Global Matrix 3.0 were collected between 2005 and 2018, and that the data available in LMICs were generally less recent than in the high-income countries. ISCOLE was a one-time cross sectional study where data were collected in each study site between 2011 and 2013, [35] with the exception of Mozambique where data were collected in 2018 [32]. The data in ICAD was obtained in 2009–2010 (ICAD 1.0) from cross-sectional and longitudinal studies from 1998 to 2009 [34]. Expansion of ICAD to include additional (more recent) waves of data and more harmonized exposure variables such as parent education, ethnicity, school travel mode/duration and car ownership is ongoing (ICAD 2.0) [37]. The GSHS does not have an established frequency, with data collection dependent on what each participating country’s resources will allow. GSHS data from Central and Eastern Europe, Latin America, and Sub-Saharan Africa were generally less recent than data available from Asia and Oceania. Finally, Marques et al. (2020), [29] Xu et al. (2020), [28] and Guthold et al. (2020) [20] were three independent analyses all performed at a single time point.

The representativeness of the data for each initiative is described in Online Supplement Material 1. In the HBSC, a nationally representative sample of 11, 13, and 15-year-olds was drawn in the majority of the participating countries and a regional sample was drawn when a national sample was not possible [27]. The GSHS used a standardized two-stage sampling design to obtain a representative sample of students in grades 9–12, [38] but several small samples were observed in some participating countries. Marques et al. (2020), [29] Guthold et al. (2020), [20] and Xu et al. (2020) [28] primarily used GSHS and HBSC data acquired through random sampling with a sample size of at least 100 individuals and representative of a national or defined subnational population. In the Global Matrix 3.0, data included was the best available data per country according to the Report Card development team and included nationally/regionally, locally, or not representative data [30]. Finally, for the two initiatives presenting device-based PA data (ISCOLE and ICAD 1.0) the samples were not intended to be nationally representative, but sampling was done in schools with an emphasis on stratification by socioeconomic status [21].^,^22).

### Comparison of physical activity assessment methods and definitions

For ISCOLE and ICAD 1.0, national PA prevalences were estimated based entirely on device-based measurements and included fewer countries than other initiatives (13 and 10, respectively) [34, 35]. The remaining initiatives were informed by self-reported PA questionnaire data, [11, 20, 28, 29, 31] with the exception of the Global Matrix 3.0, which was informed by the compilation of the best available data (reported and/or device-measured) in each participating country [30].

As described in Online Supplement Material 4, there was a clear translation protocol from English to other survey languages for the HBSC study, [27] but not for the GSHS. The validity and reliability of the moderate-to-vigorous PA (MVPA) questionnaire item of the GSHS survey and HBSC study were studied in a minority of the languages available for these initiatives. Consequently, the ability of that item to accurately detect PA across all the languages and contexts where it has been used is unknown.

Online Supplement Material 1 shows that only the Global Matrix 3.0 and ISCOLE [30, 35] provided estimates of national PA prevalence corresponding to the recent WHO recommendations for 5–17 year olds [6] (i.e., at least 60 min/day of MVPA on average). The remaining initiatives used a variety of definitions that were drawn from the previous WHO recommendations (i.e., at least 60 min of MVPA daily) [39].

In addition to inconsistencies between initiatives, some inconsistencies were also observed *within* them. The Global Matrix 3.0 was informed by various types of data across countries including device-based measurement, self-report or proxy-report questionnaire, and expert opinion; and for each of these categories, the methods varied substantially in terms of instruments, analysis, age range, sample size, and representativeness of samples [30]. However, the harmonized methods that were used to translate all of these data into a “physical activity grade” were the same across all the participating countries [30]. Similarly, the Guthold et al. and Marques et al. studies were informed by surveys that varied in terms of instruments, age range, sample size, and representativeness of samples [20, 29]. Several inconsistencies also occurred in the GSHS methods across the participating countries: some differences were observed for the PA definition, age groups, and analyses (see Online Supplement Material 3 and the full extracted datasheet available upon request). In addition, as there is no official global GSHS report, PA prevalences were only available in WHO country Fact Sheets and national GSHS Reports where the data cleaning and analysis methods were different, resulting in different national PA prevalences for the same country. In contrast, the methods used in the HBSC, ISCOLE, and ICAD 1.0 were very consistent across participating countries.

### Variation of physical activity prevalence and cross-country comparison

Online Supplement Material 2 shows that the estimated prevalence of PA for each country varied significantly by surveillance initiative. Differences in terms of study population, data collection time, PA assessment methods, and analysis across these initiatives may explain these observed variations. Despite these methodological differences, three findings were consistent across all initiatives: the generally insufficient levels of PA of children and adolescents across the world; the lower levels of PA among girls in comparison with same age boys; and the attenuation of PA levels with age.

The most concerning finding from the work presented in this paper is that cross-country comparisons varied greatly across the included initiatives. Indeed, countries with the highest and lowest prevalence of PA differed depending on the initiative. For example, the USA had one of the highest levels of PA in Guthold et al. and Marques et al., [20, 29] but one of the lowest levels in the Global Matrix 3.0, ISCOLE study and ICAD 1.0 [30, 34, 35]. A potential explanation for the observed differences between the Global Matrix 3.0 and the initiatives informed by the GSHS and HBSC could be the variety of data included in the Global Matrix 3.0. While ISCOLE and ICAD 1.0 had a low number of participating countries, they both used a standardized device-based PA assessment method and challenge the validity of the cross-country differences obtained via survey data. With no knowledge concerning the validity of the GSHS/HBSC PA questionnaire across languages and contexts, the validity of the cross-country comparisons from these initiatives is unknown. Perhaps further cultural adaptations are required for these questionnaires to accurately detect PA in different country contexts.

### Surveillance/research gaps and recommendations

The scores from the quality assessment presented in Table 1 helped to identify surveillance gaps in the PA of children and adolescents. The majority, but not all, of the initiatives presented the national PA prevalence by gender. As major gender differences are consistently observed around the world, the authors recommend that all the future surveillance initiatives should present analyses by gender, to keep monitoring this inequality.

The WHO Global Action Plan on Physical activity 2018–2030 emphasised the importance of understanding and addressing social inequalities in physical activity participation across the life spectrum [8]. With the exception of the 2017/2018 HBSC study, there was no clear reporting of PA guideline adherence by household socioeconomic status. This remains an important research and surveillance need that should be addressed in future surveillance initiatives.

There was almost no PA data available at the intercontinental level for the early years (0–4-year-olds) and children below 10 years were underrepresented compared to adolescents (11–17-year-olds) across all initiatives. Assessing movement behaviours in children younger than 10 years is challenging at the population level as valid and reliable questionnaires are lacking for this age group, [40] and there is an extra challenge to reach children aged 4 years or less as they are not typically attending school. The use of device-based assessment is a promising solution for this age group, however the cost of this type of method is likely to increase the gap between high income countries and LMICs, and low compliance with wearing devices the required amount of time has been observed [41]. A new international initiative, the International Study of Movement Behaviors in the Early Years (SUNRISE), is designed to help address this gap in the future [41]. The aims of SUNRISE are to assess the proportion of young children meeting the WHO Global guidelines, determine how movement guidelines are associated with important health, learning, and developmental outcomes in the early years, and examine variations among low-, middle- and high-income countries [41]. In addition, the Sleep and Activity Database for the Early Years (SADEY) is another ongoing project aiming to create the first harmonised database of device-based measures of young children’s PA, sedentary behaviour and sleep [42]. Finally, the Active Healthy Kids Global Alliance (AHKGA) will also encourage countries participating in the Global Matrix 4.0 and beyond to include young children in their analysis and reporting. The authors recommend that future national and international surveillance initiatives include younger children in their samples.

As shown in Table 1, the vast majority of data informing the included initiatives did not include school-aged children not attending school. Most of the PA surveillance initiatives for children and adolescents are conveniently school-based, leading to the underrepresentation of home-schooled and not-schooled children and adolescents. While this specific population is relatively small in high-income countries, it represents a larger part of the pediatric population in LMICs. It is recommended that future surveillance efforts develop recruitment methods accommodating the inclusion of this specific population in their sample, particularly in LMICs.

None of the initiatives presented in this review intentionally included children and adolescents with disability or chronic conditions. This specific population is not consistently excluded from PA surveillance initiatives; however, the data is rarely presented in a way allowing to determine differences in PA according to disability and chronic disease status. In addition, special disability schools are typically excluded from the sampling in physical activity surveillance studies. The global TEENS study was an international cross-sectional study aiming to identify the main factors associated with quality of life in 8 to 25 year-old adolescents and adults with type 1 diabetes, where PA information was collected from medical records [43]. However, no data or analysis presenting the prevalence of PA among the children and adolescents who participated in the global TEENS study are publicly available yet. Analyses on data from 15 countries that participated in the 2013/2014 HBSC were performed to compare prevalence of physically active adolescents across Europe after disaggregating for disability and adjusting for age and family affluence, but no similar analyses are available yet from the 2017/2018 HBSC study, [44] and there was no mention of including adolescents with disability or chronic condition in the HBSC protocol document [27]. Nevertheless, two AHKGA Report Cards on PA specifically targeting children and adolescents with disability or chronic condition were recently published, [45, 46] and the AHKGA is encouraging all the countries participating in the Global Matrix 4.0 to include analyses for this specific population in their Report Cards. It is recommended that future surveys include children and adolescents with disability or chronic conditions to produce needed international knowledge on this understudied population.

There was generally less data available in LMICs and the data generally was of a lower quality (i.e., smaller sample size, older data). There are several international studies/surveys including several countries within a single continent, in particular for European countries [47–50] but also some for Latin American countries [51, 52]. Overall, there are geographic inequities in PA surveillance occurring at two levels. First, there is less quality PA data in LMICs. Second, there is concern about the validity of PA questionnaires designed for use in Western high-income countries and then used in LMICs [53]. For example, is meeting the PA guidelines detected as well in African countries than in North America and Europe while using these questionnaires? What about in Asia or Latin America? These are questions that still need to be answered.

Finally, the majority of the data presented in this review were collected using self-reported questionnaires, highlighting the need for more international device-based PA data among children and adolescents. Furthermore, PA data from ISCOLE and ICAD 1.0 were collected in 2013 or before, with the exception of the ISCOLE data from Mozambique, [32] and these initiatives were not developed for nationally representative samples. Device-based measurements are hoped to become feasible for national and international PA surveillance, once further advances and consensus are achieved on translating accelerometer signals, [54] and we recommend future initiatives planning to collect device-based physical activity data to have a sampling method stratified across socioeconomic status and urban/rural settings when a nationally representative sample is not feasible. There are several ongoing surveillance projects that will help to fill the need for international device-based PA data [37, 41, 55]. An example is the International Physical activity and Environment Network (IPEN) adolescent study where 4852 adolescents (11–19-years) from 15 geographically and culturally diverse countries from six continents provided accelerometer data [55]. Similarly, papers are now being published from ICAD 2.0 which now include a wider range of harmonised variables (including data on the home environment, dietary intake and sport participation) to accompany the standardised accelerometer data. It is also anticipated that the Global Matrix 4.0, planned to be launched in 2022, will include more device-based PA data from a greater number of countries. More research is needed to develop surveillance methods allowing for the assessment of meeting the new WHO guidelines [54] while not widening the gap between high-income and LMICs.

### Summary of key recommendations

The authors recommend that national and international efforts be made to address the aforementioned surveillance and research gaps in future surveillance initiatives: they should include younger children, children from ethnic minorities and indigenous population, children with disability or chronic condition, children from urban and rural dwelling locations. In addition, analyses should be presented by gender, by household socioeconomic status, and by urban vs rural dwelling. More specifically, the authors recommend that future surveillance initiatives using report and/or device-based measurements should develop feasible and affordable methodologies to assess meeting the MVPA component of the new WHO guidelines (i.e., at least 60 min/day of MVPA on average). We also encourage future initiatives using self/proxy report measurement to have a comprehensive approach that assess all domains of physical activity for children and adolescents (i.e., leisure-time, occupational/school-based, household, travel) for a better reflection of the reality of their life across the variety of geographic, economic and cultural contexts. This information will also be key for the development of relevant and efficient interventions/policies promoting physical activity. We recommend that large international questionnaire-based surveillance initiatives use standardised device-based measurement on a portion of each of their national samples to challenge or confirm the validity of their data and facilitate international comparisons. Finally, we recommend that future surveillance initiatives employ a sampling method with stratification across socioeconomic status and urban/rural settings when a nationally representative sample is not feasible.

### Call to action

While there are no perfect PA surveillance methods for children and adolescents, this review has highlighted that the current state is discordant with its public health importance. International comparisons are study specific and vary widely. The recent publication of new WHO PA guidelines [6] for children adolescents and the aforementioned issues clearly indicate that there is an urgent need for the development of a PA measurement instrument/protocol that would be globally accepted, harmonized, and utilized. This tool should be translated and culturally adaptable in consultation with local experts before having its validity and reliability studied in a standardized way across all of its available languages.

### Strengths and limitations

This review is the first to compile and compare this amount of children and adolescent PA data, surfacing serious issues related to PA surveillance and highlighting inconsistencies, surveillance and research gaps. The authors of the present review consisted of an international panel of experts involved in many of the included initiatives (HBSC, Global Matrix 3.0, ICAD 1.0, ISCOLE) and other relevant initiatives with data not yet available (IPEN, SUNRISE, SADEY) and from both high-income countries and LMICs. All of the experts were involved in all stages of this review to prevent bias for or against a specific included initiative or missing a major source of relevant data/findings.

A limitation is that a systematic review approach was not utilized, so it was potentially missing relevant studies, and not representative of the real geographic distribution of the available surveillance initiatives. However, our approach allowed us to focus uniquely on open access/public data/findings that are most commonly referred to in major publications and generally used to inform public health institutions. In addition, the methods adopted in the present work prevented the overrepresentation of international but European/high-income country only studies. As there were no quality assessment tool suitable for the evaluation of the initiatives included in the present work, a new quality assessment tool for international surveillance of PA of children and adolescent initiatives was developed by the authors. The interpretation of the scores across the included initiatives should, however, be interpreted with caution as this tool awards more points for *clear reporting* of elements that were not necessarily relevant to all of the initiatives and presenting the PA prevalence by specific categories, rather than the actual quality of the initiatives; further refinement is warranted.

## Conclusions

The compilation and comparison of the intercontinental PA data presented in this review showed that estimated national prevalence of children and adolescents meeting PA guidelines varied significantly by surveillance initiative, however three findings were consistent across all the initiatives: the insufficient level of PA of children and adolescents across the world; the lower levels of PA among girls in comparison with same age boys; and the diminution of PA level with age. LMICs, younger children, children and adolescents not attending school, with disability or chronic conditions, and from rural areas were generally under or not represented. There are substantial inconsistencies across and within included PA surveillance initiatives, resulting in initiatives contradicting each other. Therefore, there is an urgent need for the development of a PA instrument/protocol that is globally accepted, validated, and utilized.

## Supplementary Information

**Additional file 1.** Characteristics of the included intercontinental initiatives.

**Additional file 2.** Extracted national physical activity prevalence for children and adolescents by initiative

**Additional file 3.** Summary of the characteristics of the datasets by world region for each included initiative

**Additional file 4.** Extraction of physical activity questionnaire items, translation, validity, and reliability information for the GSHS and the HBSC surveys

## Data Availability

The full extracted datasheet is available from the corresponding author by reasonable request.

## Abbreviations

PA: Physical activity
HBSC: Health Behaviour in School-aged Children
GSHS: Global School-Based Student Health Survey
ISCOLE: International Study of Childhood Obesity, Lifestyle and the Environment
ICAD: International Children’s Accelerometry Database
LMICs: Low-and middle-income countries
SGDs: Sustainable Development Goals
IPEN: International Physical activity and Environment Network
SUNRISE: International Study of Movement Behaviors in the Early Years
