# Supplementary material for: Global prevalence of physical activity for children and adolescents; inconsistencies, research gaps, and recommendations: a narrative review
Source: Int J Behav Nutr Phys Act. 2021 Jun 29;18:81. doi: 10.1186/s12966-021-01155-2 (PMC8243483; doi:10.1186/s12966-021-01155-2)
Supplement: Supplementary file 1 — Additional file 1. Characteristics of the included intercontinental initiatives. [file 12966_2021_1155_MOESM1_ESM.docx]

**Online Supplementary Material 1: Characteristics of the intercontinental initiatives included**

| **Name** | **Location** | **Target population** | **Frequency** | **Description** | **Physical activity/ inactivity definition used** | **Physical activity assessment and analysis methods** | **Representativeness of the data** |
| --- | --- | --- | --- | --- | --- | --- | --- |
| Global Matrix on physical activity for children and youth 3.0 | In the most recent cycle, 49 countries from 6 continents | 5-17-year-old children and adolescents | The Global Matrix initiative occurred every 2 years since 2014. The Global Matrix 4.0 launch is planned to take place in 2022. | The Global Matrix initiative, led by the AHKGA,^1^ brings together working groups from countries across the world who follow harmonized procedures to develop their Report Cards on PA for children and youth by grading common indicators using the best available data.^2^ | “*At least 60 mins/day of MVPA on average*”, or  “*At least 60 mins/day of MVPA on 4 or more days/week*”.^2^ | Participating countries gathered the best available data from local, national or international studies, national surveys, official reports and normative documents. Letter grades were then assigned to the PA indicator using a grading rubric to quantify PA levels (every letter grade corresponds to a specific 5-6% prevalence interval). Participating countries reported PA data that were collected using a variety of methods (self-report questionnaire, device measures) across levels.^2^ | Best available data per country: locally, nationally, or not representative. |
| Guthold et al. (2019) | 194 WHO member states, 146 countries | 11-17-years-old adolescents | This first round was performed in 2018 and updates over the next 15 years are expected. | The WHO GHO/authors compiled surveys that collected PA data among adolescents across the world and performed complex analyses to obtain an estimation of the prevalence of physical inactivity by sex and by country in 2001 and in 2016.^3^ | “Doing less than 60 minutes of physical activity on less than seven days per week”^3^ | Data were obtained from multi-country surveys (GSHS, HBSC, YRBS, NaSSDA, NNPAS, PENSE, ENSIN, ENSANUT) and national surveys using self-reported questionnaire.  Two key adjustments were applied to survey data using linear regression modelling techniques to improve comparability: a definition conversion (for surveys in which data were only reported for the definition of doing less than 60 minutes of physical activity on less than five days per week instead of seven days per week), and an adjustment for surveys that only included data from urban populations (where they estimated prevalence in rural area).  Using trend data calculated from countries with at least two comparable surveys from different years between 2001 and 2016, prevalence of insufficient physical activity was estimated for each year between 2001 and 2016 for all 146 countries, with a multilevel mixed-effects linear regression model. This model included a random slope on year, a random intercept for each country, fixed effects for country urbanisation, and location within nine previously defined regions.^3^ | Data were acquired through random sampling with a sample size of at least 100 individuals and representative of a national or defined subnational population.^3^ |
| GSHS, reported in Official WHO Fact Sheets or official WHO GSHS country reports | WHO fact sheets are available for 87 countries from Central and eastern Europe; Central Asia, Middle East, and North Africa; East and Southeast Asia; Latin America and Caribbean; Oceania; South Asia; Sub-Saharan Africa.^4^ | 13–18-year-old students | No established frequency, participating countries are encouraged to collect data as often as resources allow. | The GSHS is a school-based survey using a self-administered questionnaire to evaluate young people's health behaviour and protective factors related to the leading causes of morbidity and mortality among children and adults worldwide.^5^ It was developed by the  WHO and CDC in collaboration with UNICEF, UNESCO,  and UNAIDS.^6^ | “*Percentage of students who were physically active for a total of at least 60 minutes per day on five or more days during the past seven days*” or “*Percentage of students who were physically active for a total of at least 60 minutes per day on seven days during the past seven days*” | The Official GSHS questionnaire used the following item to assess PA: “*During the past 7 days, on how many days were you physically active for a total of at least 60 minutes per day? Add up all the time you spent in physical activity each day*.” With eight possible answers (from “*0 days*” to “*7 days*”)  All data processing (scanning, cleaning, editing, and weighting) were conducted at CDC. Two workshops were provided to the Survey Coordinators from each participating country. One to build the capacity of Survey Coordinators to implement the survey in their country following common sampling and survey administration procedures that ensure the surveys are standardized and comparable across countries and that data are of the highest quality; and the second to build the capacity of Survey Coordinators to conduct data analysis and generate a country-specific report and fact sheet using *Epi-Info software* provided to them.^7^ | The GSHS used a standardized two-stage sampling design to obtain a representative sample of students in grades 9–12.^8^ |
| 2017/2018 Health Behaviour in School-Aged Children (HBSC) Survey (official international report) | In the most recent HBSC cycle, 48 countries and regions across Europe, Middle East, and North America^9^ | 11-, 13-, and 15-year-old school students^10^ | The HBSC survey occurred every four years since 1982^10^ | The HBSC is a school-based survey, where a standardized self-reported questionnaire is administered to a nationally representative sample of 11-, 13- and 15-year-olds within the classroom setting. Among the purposes of the HBSC were to: determine the proportion of adolescents who meet the current recommendation for daily PA, by age and gender; determine the frequency of leisure-time VPA; follow and describe trends in adolescent PA; identify correlates and determinants of MVPA and VPA; investigate health outcomes associated with PA and physical inactivity; and explore the clustering of energy balance-related behaviours (PA, screen-time behaviours, sleep and dietary patterns).^10^ | “*At least 60 minutes of MVPA daily*”^9^ | The 2017/2018 HBSC included two mandatory items in all participating countries/ regions to evaluate PA: “*Over the past 7 days, on how many days were you physically active for a total of at least 60 minutes per day? Please add up all the time you spent in physical activity each day*.” With eight possible answers (from “*0 days*” to “*7 days*”); and “*Outside school hours: how often do you usually exercise in your free time so much that you get out of breath or sweat?”* with six possible answers: *“Every day/ 4 to 6 times a week/ 2 to 3 times a week/ Once a week/ Once a month/ Less than once a month/ Never*”.^10^  In the official 2017/2018 HBSC international report, prevalence of reporting at least 60 minutes of MVPA daily were presented according to age, gender and family affluence.^9^ | A nationally representative sample was drawn in the majority of countries/regions; where a national sample was not possible, a regional  sample was drawn (the minimum size of the total population for regional samples should be 1 million). A census among the relevant age groups was taken in countries/regions where the population was sufficiently small, with all classes of young people in the relevant age groups  being surveyed.^10^ |
| International Children's Accelerometry Database (ICAD) 1.0 | Datasets are available for 10 countries: Australia, Brazil, Belgium, Denmark, UK, Estonia, Norway, Portugal, Switzerland, United States | 3-18-year-old children and adolescents | Data from cross sectional and longitudinal studies (1998-2009) were pooled, reduced and harmonized between 2008-2010. | The ICAD was established to pool data on device measured PA from studies using the same type of accelerometer (Actigraph) worn at the waist. Investigators from 20 studies with a sample size >400 (for school aged studies) agreed to share raw accelerometery files, and standardized data reduction were performed to create comparable outcome variables across studies.^11^ | “*Achieving ≥60 min of MVPA each measurement day*”.^12^ | PA was assessed with *Actigraph* accelerometers waist-worn for at least three days. Data files were reintegrated to a 60s epoch and processed using *KineSoft v3.3.20*. MVPA was defined as >2296 cpm. Analyses were restricted to 9-10- and 12-13-year-olds participants.^12^ | Seven countries had MVPA data for ages 9-10 while only 4 (Brazil, USA, England and Australia) had MVPA data for ages 12-13.^12^ National/regional representativeness of samples was not a requirement for inclusion. |
| International Study of Childhood Obesity, Lifestyle and the Environment (ISCOLE) | 13 countries from five major geographic regions of the world (Europe, Africa, the Americas, South-East Asia, and the Western Pacific) | 9-11-year-old children | ISCOLE is a cross sectional study where data were collected in each study site between 2011 and 2013,^13^ with the exception of Mozambique where data were collected in 2018. | ISCOLE aimed to determine the relationships between lifestyle behaviours and obesity, and to study the influence of additional characteristics such as behavioural settings, physical, social and policy environments, on the observed relationships.^13^ | ≥60 mins/day of MVPA on average^14^ | PA was assessed with *Actigraph GT3X+* accelerometers waist worn for at least seven days, including two weekend days. Data were collected at a sampling rate of 80 Hz, downloaded in 1-s epochs with the low frequency extension filter using the *ActiLife software version 5.6* or higher (ActiGraph LLC, Pensacola, FL, U.S.A). Data were later reintegrated to 15-s and 60-s epochs for the different analyses. MVPA was defined as >574 counts per 15s.^13^ | The within-site samples were not intended to be nationally representative. The sampling was done in schools, where the emphasis was put on stratification by socioeconomic status in order to maximize variability within sites.^13^ |
| Marques et al. (2020) | 105 countries across eight regions: Central and Eastern Europe, Central Asia/Middle East and North Africa, East and Southeast Asia, High-income western countries, Oceania, Sub-Sharan Africa, South Asia and Latin America/ Caribbean.^15^ | 11-17-years-old adolescents^15^ | Single study | The aim of this paper was to present worldwide, national, and regional prevalence of PA participation according to its frequency in adolescents aged 11-17 years. The study used cross-sectional survey data from multiple different surveys (HBSC, GSHS, YRBS, PENSE, ENSANUT) that collected self-reported PA prevalence among adolescents.^15^ | Physically active for a total of at least 60 minutes per day over the past 7 days. | According to the authors, the same unique question was used across the included surveys to assess the adolescents’ PA levels: “*During the past 7 days, on how many days were you physically active for a total of at least 60 minutes per day?*”. Answers were given on an 8-point scale (0 = none to 7 = daily). The prevalence of PA (replying “7”) was calculated stratified by sex, age (11-12, 13-15 and 16-17), eight world regions, Human Development Index, and by country.^15^ | Dataset included were collected from officially representative national sample sizes of at least 100 adolescents per country.^15^ |
| Xu et al. 2020 | 54 low to medium income countries from 5 regions: Africa, Americas, Eastern Mediterranean, Southeast Asia and Western Pacific^16^ | 12-15 years-old adolescents^16^ | No established frequency; single study | The aim of this study was to describe and compare the separate and combined prevalence of physical activity, active transportation, physical education, and sedentary behavior among adolescents aged 12-15 in low- and middle-income countries using GSHS data.^16^ | “*Adolescents who were physically active for at least 1 hour per day were considered to engage in physical activity*”.^16^ | The official GSHS questionnaire uses the following question: “*During the past 7 days, on how many days were you physically active for a total of at least 60 mins per day?*”.  Estimations of PA prevalence values and 95% confidence intervals were presented for the 12-15 by country and by sex and age, calculated using the *SAS Surveymeans* procedure. Authors added weights, stratum, and a primary sampling unit to every student record in the GSHS data file to reflect the weighting process and the two-stage sampling design in their analysis.^16^ | The GSHS uses a standardized two-stage sampling design to obtain a representative sample of students in grades 9–12.^8^ |

Notes: PA = physical activity; AHKGA = Active Healthy Kids Global Alliance; cpm = count per minute; MVPA = moderate-to-vigorous physical activity; HBSC = Health Behaviour in School-aged Children; ICAD = International Children's Accelerometry Database; ISCOLE = International Study of Childhood Obesity, Lifestyle and the Environment; WHO = World Health Organisation; GSHS = Global School-Based Student Health Survey; YRBS = Youth Risk Behavior Surveillance; PENSE = Pesquisa Nacional de Saúde do Escolar; ENSANUT = Encuesta Nacional de Salud y Nutrición; GHO = Global Health Observatory; CDC = Center for Disease Control and Prevention; UNICEF = United Nations Children's Fund ; UNESCO = United Nations Educational, Scientific and Cultural Organisation; UNAIDS = Joint United Nations Programme on HIV and AIDS; NaSSDA = National Secondary Students' Diet and Activity; NNPAS = National Nutrition and Physical Activity Survey; ENSIN = Encuesta Nacional de Situación Nutricional.

**References**

1. Active Healthy Kids Global Alliance. About » Active Healthy Kids Global Alliance. https://www.activehealthykids.org/about/. Published 2018. Accessed January 31, 2019.

2. Aubert S, Barnes JD, Abdeta C, et al. Global Matrix 3.0 Physical Activity Report Card Grades for Children and Youth: Results and Analysis from 49 Countries. *J Phys Act Heal*. 2018;15(S2):S251-S273. doi:10.1123/jpah.2018-0472

3. Guthold R, Stevens GA, Riley LM, Bull FC. Global trends in insufficient physical activity among adolescents: a pooled analysis of 298 population-based surveys with 1·6 million participants. *Lancet Child Adolesc Heal*. 2019;4(1):23-35. doi:https://doi.org/10.1016/S2352-4642(19)30323-2

4. NCDs | Global school-based student health survey (GSHS). *WHO*. 2020. http://www.who.int/ncds/surveillance/gshs/datasets/en/. Accessed August 7, 2020.

5. World Health Organization. Global school-based student health survey (GSHS). WHO. https://www.who.int/ncds/surveillance/gshs/en/. Published 2018. Accessed January 25, 2019.

6. CDC Global School-based Student Health Survey (GSHS). https://www.cdc.gov/GSHS/. Accessed August 7, 2020.

7. NCDs | Global school-based student health survey (GSHS) capacity building and training. *WHO*. 2017. http://www.who.int/ncds/surveillance/gshs/training/en/. Accessed August 7, 2020.

8. NCDs | Global school-based student health survey (GSHS) purpose and methodology. *WHO*. 2020. http://www.who.int/ncds/surveillance/gshs/methodology/en/. Accessed August 7, 2020.

9. World Health Organization. *Spotlight on Adolescent Health and Well-Being. Findings from the 2017/2018 Health Behaviour in School-Aged Children (‎‎HBSC)‎‎ Survey in Europe and Canada. International Report. Volume 2. Key Data.* Geneva; 2020. https://apps.who.int/iris/handle/10665/332104.

10. Currie C, Inchley J, Molcho M, Lenzi M, Veselska Z, Wild F. Health Behaviour in School-aged Children (HBSC) study protocol: Background, methodology and mandatory items for the 2013/14 survey. 2014. https://researchonline.gcu.ac.uk/en/publications/health-behaviour-in-school-aged-children-hbsc-study-protocol-back. Accessed August 7, 2020.

11. Sherar LB, Griew P, Esliger DW, et al. International children’s accelerometry database (ICAD): Design and methods. *BMC Public Health*. 2011;11(1):485. doi:10.1186/1471-2458-11-485

12. Cooper AR, Goodman A, Page AS, et al. Objectively measured physical activity and sedentary time in youth: the International children’s accelerometry database (ICAD). *Int J Behav Nutr Phys Act*. 2015;12(1):113. doi:10.1186/s12966-015-0274-5

13. Katzmarzyk PT, Barreira T V, Broyles ST, et al. The International Study of Childhood Obesity, Lifestyle and the Environment (ISCOLE): design and methods. *BMC Public Health*. 2013;13(1):900. doi:10.1186/1471-2458-13-900

14. Roman-Viñas B, Chaput JP, Katzmarzyk PT, et al. Proportion of children meeting recommendations for 24-hour movement guidelines and associations with adiposity in a 12-country study. *Int J Behav Nutr Phys Act*. 2016;13(1):123. doi:10.1186/s12966-016-0449-8

15. Marques A, Henriques-Neto D, Peralta M, et al. Prevalence of Physical Activity among Adolescents from 105 Low, Middle, and High-Income Countries. *Int J Environ Res Public Health*. 2020;17(9):3145. doi:10.3390/ijerph17093145

16. Xu G, Sun N, Li L, et al. Physical behaviors of 12-15 year-old adolescents in 54 low-and middle-income countries: Results from the Global School-based Student Health Survey. *J Glob Health*. 2020;10(1). doi:10.7189/jogh.10.010423
