# Supplementary material for: Global prevalence of physical activity for children and adolescents; inconsistencies, research gaps, and recommendations: a narrative review
Source: Int J Behav Nutr Phys Act. 2021 Jun 29;18:81. doi: 10.1186/s12966-021-01155-2 (PMC8243483; doi:10.1186/s12966-021-01155-2)
Supplement: Supplementary file 2 — Additional file 2. Extracted national physical activity prevalence for children and adolescents by initiative [file 12966_2021_1155_MOESM2_ESM.docx]

**Online Supplement Material 2**: Extracted national physical activity prevalence for children and adolescents by initiative.

|  | **Prevalence of physical activity among children and/or adolescents** | | | | | | | | | | | | | | | | | | | | | |
| --- | --- | --- | --- | --- | --- | --- | --- | --- | --- | --- | --- | --- | --- | --- | --- | --- | --- | --- | --- | --- | --- | --- |
|  | **Guthold et al.,2020^a^** | | | **Global Matrix 3.0^b^** | **2017/2018 HBSC^c^** | | | **GSHS survey (Official WHO country Fact Sheets or GSHS national reports)^c^** | | | | **ISCOLE^d^** | **ICAD 1.0^c^** | | | **Marques et al., 2020^e^** | | | **Xu et al., 2020^c^** | | | |
| **Countries** | **Overall** | **Boys** | **Girls** | **Overall** | **Age** | **Boys** | **Girls** | **Age** | **Overall** | **Boys** | **Girls** | **Overall** | **Age** | **Boys** | **Girls** | **Overall** | **Boys** | **Girls** | **Age** | **Overall** | **Boys** | **Girls** |
| **Central and Eastern Europe** |  |  |  |  |  |  |  |  |  |  |  |  |  |  |  |  |  |  |  |  |  |  |
| Albania | 26.1 | 32.4 | 19.2 |  | 11 | 26 | 23 |  |  |  |  |  |  |  |  | 28 |  |  |  |  |  |  |
|  |  |  |  |  | 13 | 34 | 24 |  |  |  |  |  |  |  |  |  |  |  |  |  |  |  |
|  |  |  |  |  | 15 | 22 | 8 |  |  |  |  |  |  |  |  |  |  |  |  |  |  |  |
| Bulgaria | 26.7 | 33 | 19.9 | 34%–39% | 11 | 26 | 25 |  |  |  |  |  |  |  |  | 28.6 |  |  |  |  |  |  |
|  |  |  |  |  | 13 | 26 | 21 |  |  |  |  |  |  |  |  |  |  |  |  |  |  |  |
|  |  |  |  |  | 15 | 23 | 17 |  |  |  |  |  |  |  |  |  |  |  |  |  |  |  |
| Croatia | 23.2 | 29.9 | 16 |  | 11 | 31 | 25 |  |  |  |  |  |  |  |  | 25.6 |  |  |  |  |  |  |
|  |  |  |  |  | 13 | 27 | 22 |  |  |  |  |  |  |  |  |  |  |  |  |  |  |  |
|  |  |  |  |  | 15 | 21 | 13 |  |  |  |  |  |  |  |  |  |  |  |  |  |  |  |
| Czech Republic | 22.6 | 26.9 | 18 | 27%–33% | 11 | 26 | 19 |  |  |  |  |  |  |  |  | 21.3 |  |  |  |  |  |  |
|  |  |  |  |  | 13 | 22 | 14 |  |  |  |  |  |  |  |  |  |  |  |  |  |  |  |
|  |  |  |  |  | 15 | 17 | 10 |  |  |  |  |  |  |  |  |  |  |  |  |  |  |  |
| Estonia | 15.9 | 19.4 | 12.3 | 20%–26% | 11 | 20 | 18 |  |  |  |  |  | 9-10 | 26 | 18 | 16.4 |  |  |  |  |  |  |
|  |  |  |  |  | 13 | 16 | 12 |  |  |  |  |  |  |  |  |  |  |  |  |  |  |  |
|  |  |  |  |  | 15 | 16 | 11 |  |  |  |  |  |  |  |  |  |  |  |  |  |  |  |
| Hungary | 20.5 | 26.5 | 14.2 |  | 11 | 31 | 24 |  |  |  |  |  |  |  |  | 16.4 |  |  |  |  |  |  |
|  |  |  |  |  | 13 | 20 | 15 |  |  |  |  |  |  |  |  |  |  |  |  |  |  |  |
|  |  |  |  |  | 15 | 18 | 11 |  |  |  |  |  |  |  |  |  |  |  |  |  |  |  |
| Latvia | 19.9 | 23.8 | 15.8 |  | 11 | 23 | 20 |  |  |  |  |  |  |  |  | 18.4 |  |  |  |  |  |  |
|  |  |  |  |  | 13 | 23 | 12 |  |  |  |  |  |  |  |  |  |  |  |  |  |  |  |
|  |  |  |  |  | 15 | 20 | 14 |  |  |  |  |  |  |  |  |  |  |  |  |  |  |  |
| Lithuania | 19.9 | 24 | 15.5 | 40%–46% | 11 | 24 | 22 |  |  |  |  |  |  |  |  |  |  |  |  |  |  |  |
|  |  |  |  |  | 13 | 20 | 14 |  |  |  |  |  |  |  |  |  |  |  |  |  |  |  |
|  |  |  |  |  | 15 | 17 | 12 |  |  |  |  |  |  |  |  |  |  |  |  |  |  |  |
| Republic of Moldova | 24.3 | 26.7 | 21.8 |  | 11 | 21 | 17 |  |  |  |  |  |  |  |  |  |  |  |  |  |  |  |
|  |  |  |  |  | 13 | 16 | 10 |  |  |  |  |  |  |  |  |  |  |  |  |  |  |  |
|  |  |  |  |  | 15 | 13 | 11 |  |  |  |  |  |  |  |  |  |  |  |  |  |  |  |
| North Macedonia | 21.6 | 26.5 | 16.5 |  | 11 | 42 | 35 | 13 and older | 15.2 | 17.2 | 13 |  |  |  |  | 26.9 |  |  |  |  |  |  |
|  |  |  |  |  | 13 | 38 | 25 | 13-15 | 16.1 |  |  |  |  |  |  |  |  |  |  |  |  |  |
| North Macedonia |  |  |  |  | 15 | 31 | 17 | 16 and older | 11.1 |  |  |  |  |  |  |  |  |  |  |  |  |  |
| Poland | 21.2 | 26.3 | 15.8 | 20%–26% | 11 | 24 | 22 |  |  |  |  |  |  |  |  | 24 |  |  |  |  |  |  |
|  |  |  |  |  | 13 | 16 | 15 |  |  |  |  |  |  |  |  |  |  |  |  |  |  |  |
|  |  |  |  |  | 15 | 18 | 9 |  |  |  |  |  |  |  |  |  |  |  |  |  |  |  |
| Romania | 20.5 | 27.3 | 13.3 |  | 11 | 21 | 15 |  |  |  |  |  |  |  |  | 21.3 |  |  |  |  |  |  |
|  |  |  |  |  | 13 | 16 | 9 |  |  |  |  |  |  |  |  |  |  |  |  |  |  |  |
|  |  |  |  |  | 15 | 16 | 7 |  |  |  |  |  |  |  |  |  |  |  |  |  |  |  |
| Russia | 15.5 | 19.1 | 11.7 |  | 11 | 26 | 18 |  |  |  |  |  |  |  |  | 17.9 |  |  |  |  |  |  |
|  |  |  |  |  | 13 | 18 | 10 |  |  |  |  |  |  |  |  |  |  |  |  |  |  |  |
|  |  |  |  |  | 15 | 16 | 9 |  |  |  |  |  |  |  |  |  |  |  |  |  |  |  |
| Serbia |  |  |  |  | 11 | 45 | 36 |  |  |  |  |  |  |  |  |  |  |  |  |  |  |  |
|  |  |  |  |  | 13 | 44 | 25 |  |  |  |  |  |  |  |  |  |  |  |  |  |  |  |
|  |  |  |  |  | 15 | 39 | 18 |  |  |  |  |  |  |  |  |  |  |  |  |  |  |  |
| Slovakia | 28.5 | 34.5 | 22.2 |  | 11 | 28 | 26 |  |  |  |  |  |  |  |  | 25.3 |  |  |  |  |  |  |
|  |  |  |  |  | 13 | 29 | 17 |  |  |  |  |  |  |  |  |  |  |  |  |  |  |  |
|  |  |  |  |  | 15 | 24 | 12 |  |  |  |  |  |  |  |  |  |  |  |  |  |  |  |
| Slovenia | 20 | 25.2 | 14.4 | 80%–86% | 11 | 31 | 22 |  |  |  |  |  |  |  |  | 18.5 |  |  |  |  |  |  |
|  |  |  |  |  | 13 | 28 | 17 |  |  |  |  |  |  |  |  |  |  |  |  |  |  |  |
|  |  |  |  |  | 15 | 24 | 11 |  |  |  |  |  |  |  |  |  |  |  |  |  |  |  |
| Ukraine | 20.1 | 25.3 | 14.6 |  | 11 | 34 | 27 |  |  |  |  |  |  |  |  | 26.3 |  |  |  |  |  |  |
|  |  |  |  |  | 13 | 29 | 20 |  |  |  |  |  |  |  |  |  |  |  |  |  |  |  |
|  |  |  |  |  | 15 | 28 | 16 |  |  |  |  |  |  |  |  |  |  |  |  |  |  |  |
|  |  |  |  |  |  |  |  |  |  |  |  |  |  |  |  |  |  |  |  |  |  |  |
| **Central Asia, Middle East, and North Africa** |  |  |  |  |  |  |  |  |  |  |  |  |  |  |  |  |  |  |  |  |  |  |
| Algeria | 16.2 | 23.6 | 8.7 |  |  |  |  |  |  |  |  |  |  |  |  | 15.1 |  |  |  |  |  |  |
| Armenia | 22.3 | 27 | 17.2 |  | 11 | 35 | 30 |  |  |  |  |  |  |  |  | 22.9 |  |  |  |  |  |  |
|  |  |  |  |  | 13 | 30 | 24 |  |  |  |  |  |  |  |  |  |  |  |  |  |  |  |
|  |  |  |  |  | 15 | 26 | 15 |  |  |  |  |  |  |  |  |  |  |  |  |  |  |  |
| Azerbaijan |  |  |  |  | 11 | 21 | 17 |  |  |  |  |  |  |  |  |  |  |  |  |  |  |  |
|  |  |  |  |  | 13 | 23 | 10 |  |  |  |  |  |  |  |  |  |  |  |  |  |  |  |
| Azerbaijan |  |  |  |  | 15 | 18 | 14 |  |  |  |  |  |  |  |  |  |  |  |  |  |  |  |
| Bahrain | 19 | 25 | 12.6 |  |  |  |  | 13-17 | 19.5 | 26 | 12.6 |  |  |  |  |  |  |  |  |  |  |  |
|  |  |  |  |  |  |  |  | 13-15 | 20.7 | 26.9 | 13.9 |  |  |  |  |  |  |  |  |  |  |  |
|  |  |  |  |  |  |  |  | 16-17 | 16.7 | 23.7 | 9.1 |  |  |  |  |  |  |  |  |  |  |  |
| Egypt | 12.5 | 17.9 | 6.9 |  |  |  |  | 13-15 | 16.5 | 23 | 10.3 |  |  |  |  | 14.5 |  |  | 12-15 | 12.6 | 18.6 | 7 |
|  |  |  |  |  |  |  |  |  |  |  |  |  |  |  |  |  |  |  | 12-13 | 13.5 |  |  |
|  |  |  |  |  |  |  |  |  |  |  |  |  |  |  |  |  |  |  | 14-15 | 11.6 |  |  |
| Georgia |  |  |  |  | 11 | 25 | 16 |  |  |  |  |  |  |  |  |  |  |  |  |  |  |  |
|  |  |  |  |  | 13 | 24 | 18 |  |  |  |  |  |  |  |  |  |  |  |  |  |  |  |
|  |  |  |  |  | 15 | 26 | 19 |  |  |  |  |  |  |  |  |  |  |  |  |  |  |  |
| Iraq | 15 | 20.1 | 9.5 |  |  |  |  | 13-15 | 20 | 25.3 | 13.6 |  |  |  |  | 14.6 |  |  | 12-15 | 14.7 | 19.5 | 8.9 |
|  |  |  |  |  |  |  |  |  |  |  |  |  |  |  |  |  |  |  | 12-13 | 16.8 |  |  |
|  |  |  |  |  |  |  |  |  |  |  |  |  |  |  |  |  |  |  | 14-15 | 13.7 |  |  |
| Jordan | 15.2 | 18.7 | 11.6 |  |  |  |  | 13-15 | 11.2 | 14.7 | 7.9 |  |  |  |  |  |  |  |  |  |  |  |
| Kazakhstan |  |  |  |  | 11 | 37 | 35 |  |  |  |  |  |  |  |  |  |  |  |  |  |  |  |
|  |  |  |  |  | 13 | 37 | 35 |  |  |  |  |  |  |  |  |  |  |  |  |  |  |  |
|  |  |  |  |  | 15 | 35 | 27 |  |  |  |  |  |  |  |  |  |  |  |  |  |  |  |
| Kuwait | 15.7 | 20.6 | 10 |  |  |  |  | 13-17 | 15.6 | 18.1 | 13.1 |  |  |  |  | 15.5 |  |  | 12-15 | 16.8 | 18.9 | 14.7 |
|  |  |  |  |  |  |  |  | 13-15 | 17.1 | 19 | 15.1 |  |  |  |  |  |  |  | 12-13 | 19.1 |  |  |
|  |  |  |  |  |  |  |  | 16-17 | 13.4 | 16.8 | 10 |  |  |  |  |  |  |  | 14-15 | 15.9 |  |  |
| Lebanon | 17.9 | 24 | 12.1 | 27%–33% |  |  |  | 13-17 | 13.2 | 18.6 | 8.7 |  |  |  |  | 24.6 |  |  | 12-15 | 23.4 | 31.2 | 16.6 |
|  |  |  |  |  |  |  |  | 13-15 | 15.6 | 21.4 | 10.5 |  |  |  |  |  |  |  | 12-13 | 26 |  |  |
|  |  |  |  |  |  |  |  | 16-17 | 9.2 | 13.4 | 5.8 |  |  |  |  |  |  |  | 14-15 | 21.8 |  |  |
| Libya | 16.8 | 22 | 11.4 |  |  |  |  | 13-15 | 15.8 | 20.9 | 10.8 |  |  |  |  |  |  |  |  |  |  |  |
| Mongolia | 21.3 | 25.8 | 16.6 |  |  |  |  | 13-17 | 36.4 | 40.7 | 32.2 |  |  |  |  | 25.2 |  |  | 12-15 | 26.9 | 31.5 | 22.4 |
|  |  |  |  |  |  |  |  | 13-15 | 37.6 | 40.6 | 34.5 |  |  |  |  |  |  |  | 12-13 | 28.5 |  |  |
|  |  |  |  |  |  |  |  | 16-17 | 34 | 40.8 | 28.2 |  |  |  |  |  |  |  | 14-15 | 255 |  |  |
| Morocco | 12.7 | 15.4 | 9.9 |  |  |  |  | 13-17 | 11 | 13.5 | 8.2 |  |  |  |  | 13.1 |  |  | 12-15 | 12.4 | 14.7 | 10.1 |
|  |  |  |  |  |  |  |  | 13-15 | 11.5 | 13.9 | 9 |  |  |  |  |  |  |  | 12-13 | 11.9 |  |  |
|  |  |  |  |  |  |  |  | 16-17 | 10.1 | 12.8 | 6.7 |  |  |  |  |  |  |  | 14-15 | 12.8 |  |  |
| Occupied Palestinian territory including Jerusalem | 16 | 20.5 | 11.4 |  |  |  |  |  |  |  |  |  |  |  |  | 15.7 |  |  |  |  |  |  |
| Gaza Strip |  |  |  |  |  |  |  | 13-15 | 24.2 | 29.3 | 18.6 |  |  |  |  |  |  |  |  |  |  |  |
| West Bank |  |  |  |  |  |  |  | 13-15 | 18.3 | 22.8 | 13.5 |  |  |  |  |  |  |  |  |  |  |  |
| Oman | 16.2 | 21.7 | 10.2 |  |  |  |  | 13-17 | 11.7 | 15.4 | 8.3 |  |  |  |  | 11.7 |  |  | 12-15 | 13 | 16.4 | 10.1 |
|  |  |  |  |  |  |  |  | 13-15 | 13.2 | 16.8 | 10.2 |  |  |  |  |  |  |  | 12-13 | 14.2 |  |  |
|  |  |  |  |  |  |  |  | 16-17 | 9.8 | 13.9 | 5.9 |  |  |  |  |  |  |  | 14-15 | 12.6 |  |  |
| Qatar | 11.8 | 13.7 | 9.1 | 27%–33% |  |  |  | 13-15 | 15 | 19.9 | 10.2 |  |  |  |  |  |  |  | 12-15 | 10.5 | 12.8 | 8.6 |
|  |  |  |  |  |  |  |  |  |  |  |  |  |  |  |  |  |  |  | 12-13 | 12.7 |  |  |
|  |  |  |  |  |  |  |  |  |  |  |  |  |  |  |  |  |  |  | 14-15 | 8.1 |  |  |
| Syria | 12.5 | 15.9 | 8.9 |  |  |  |  | 13-15 | 15.1 | 19.1 | 10.8 |  |  |  |  | 10.6 |  |  | 12-15 | 11.2 | 14.7 | 7.6 |
|  |  |  |  |  |  |  |  |  |  |  |  |  |  |  |  |  |  |  | 12-13 | 10.6 |  |  |
|  |  |  |  |  |  |  |  |  |  |  |  |  |  |  |  |  |  |  | 14-15 | 11.8 |  |  |
| Tunisia | 18.5 | 25.1 | 11.6 |  |  |  |  | 13-15 | 18.5 | 26.2 | 11 |  |  |  |  |  |  |  |  |  |  |  |
| Turkey | 18.7 | 23.4 | 13.9 |  |  |  |  |  |  |  |  |  |  |  |  |  |  |  |  |  |  |  |
| United Arab Emirates | 18.1 | 22.1 | 13.1 | <20% |  |  |  | 13-17 | 15.7 | 20.5 | 11.2 |  |  |  |  | 18 |  |  | 12-15 | 17.1 | 22.7 | 13.5 |
|  |  |  |  |  |  |  |  | 13-15 | 16.7 | 21.6 | 12.1 |  |  |  |  |  |  |  | 12-13 | 18.8 |  |  |
|  |  |  |  |  |  |  |  | 16-17 | 14.1 | 18.8 | 9.7 |  |  |  |  |  |  |  | 14-15 | 16.3 |  |  |
| Uzbekistan |  |  |  |  |  |  |  |  |  |  |  |  |  |  |  |  |  |  |  |  |  |  |
| Yemen | 13.6 | 16.7 | 10.4 |  |  |  |  | 13-17 | 11.5 | 14.3 | 8.6 |  |  |  |  | 11.9 |  |  |  |  |  |  |
|  |  |  |  |  |  |  |  | 13-15 | 12.3 | 15.4 | 8.5 |  |  |  |  |  |  |  |  |  |  |  |
|  |  |  |  |  |  |  |  | 16-17 | 10.2 | 12 | 8.6 |  |  |  |  |  |  |  |  |  |  |  |
|  |  |  |  |  |  |  |  |  |  |  |  |  |  |  |  |  |  |  |  |  |  |  |
| **East and Southeast Asia** |  |  |  |  |  |  |  |  |  |  |  |  |  |  |  |  |  |  |  |  |  |  |
| Brunei | 12.9 | 18.9 | 6.5 |  |  |  |  | 13-17 | 12.7 | 19.7 | 5.5 |  |  |  |  | 12.1 |  |  | 12-15 | 11.5 | 18.4 | 5 |
|  |  |  |  |  |  |  |  | 13-15 | 11.9 | 19.1 | 5.2 |  |  |  |  |  |  |  | 12-13 | 10.7 |  |  |
|  |  |  |  |  |  |  |  | 16-17 | 14.5 | 21.2 | 6.4 |  |  |  |  |  |  |  | 14-15 | 11.8 |  |  |
| Cambodia | 8.4 | 10.2 | 6.6 |  |  |  |  | 13-17 | 9.8 | 11.4 | 8.3 |  |  |  |  | 7.3 |  |  | 12-15 | 6.3 | 8.2 | 4.7 |
|  |  |  |  |  |  |  |  | 13-15 | 9.3 | 10.6 | 8 |  |  |  |  |  |  |  | 12-13 | 6.3 |  |  |
|  |  |  |  |  |  |  |  | 16-17 | 10.8 | 12.6 | 8.7 |  |  |  |  |  |  |  | 14-15 | 6.4 |  |  |
| China | 15.7 | 19.9 | 10.9 | <20% |  |  |  | 13-15 | 12.2 | 15.7 | 8.9 | 15.1 |  |  |  |  |  |  |  |  |  |  |
| Taiwan | 15.6 | 20.9 | 10.1 | <20% |  |  |  | 13-15 | 28.7 | 36.6 | 20.5 |  |  |  |  |  |  |  |  |  |  |  |
| Hong Kong |  |  |  | 40%–46% |  |  |  |  |  |  |  |  |  |  |  |  |  |  |  |  |  |  |
| Indonesia | 13.6 | 14.6 | 12.6 |  |  |  |  | 13-17 | 12.9 | 13.4 | 12.3 |  |  |  |  |  |  |  | 12-15 | 11.9 | 12.7 | 11.3 |
|  |  |  |  |  |  |  |  | 13-15 | 12.8 | 13.4 | 12.2 |  |  |  |  |  |  |  | 12-13 | 11.4 |  |  |
|  |  |  |  |  |  |  |  | 16-17 | 13.1 | 13.7 | 12.5 |  |  |  |  |  |  |  | 14-15 | 12.5 |  |  |
| Laos | 15.6 | 22 | 9 |  |  |  |  | 13-17 | 16.8 | 24 | 8.6 |  |  |  |  | 15.2 |  |  | 12-15 | 16.2 | 24.6 | 8.8 |
|  |  |  |  |  |  |  |  | 13-15 | 16.3 | 24.5 | 8.8 |  |  |  |  |  |  |  | 12-13 | 17 |  |  |
|  |  |  |  |  |  |  |  | 16-17 | 17.1 | 23.8 | 8.4 |  |  |  |  |  |  |  | 14-15 | 16.2 |  |  |
| Malaysia | 13.8 | 19.4 | 8.6 |  |  |  |  | 13-17 | 22.8 | 30.2 | 15.3 |  |  |  |  | 14.9 |  |  | 12-15 | 13.8 | 19.4 | 8.3 |
|  |  |  |  |  |  |  |  | 13-15 | 21.7 | 28.3 | 15.2 |  |  |  |  |  |  |  | 12-13 | 13.6 |  |  |
|  |  |  |  |  |  |  |  | 16-17 | 24.5 | 33.3 | 15.5 |  |  |  |  |  |  |  | 14-15 | 13.9 |  |  |
| Maldives | 18.1 | 22.1 | 13.9 |  |  |  |  | 13-15 | 27 | 29.5 | 24.5 |  |  |  |  |  |  |  |  |  |  |  |
| Myanmar | 13.2 | 15.9 | 10.4 |  |  |  |  | 13-17 | 10.3 | 12.8 | 8.2 |  |  |  |  |  |  |  |  |  |  |  |
|  |  |  |  |  |  |  |  | 13-15 | 10.1 | 12.5 | 8.1 |  |  |  |  |  |  |  |  |  |  |  |
|  |  |  |  |  |  |  |  | 16-17 | 11.2 | 14.7 | 8.3 |  |  |  |  |  |  |  |  |  |  |  |
| Philippines | 6.6 | 7.2 | 5.9 |  |  |  |  | 13-17 | 7.6 | 8.2 | 7 |  |  |  |  | 7.7 |  |  | 12-15 | 7.3 | 7.8 | 6.9 |
|  |  |  |  |  |  |  |  | 13-15 | 7.3 | 7.7 | 6.9 |  |  |  |  |  |  |  | 12-13 | 7.7 |  |  |
|  |  |  |  |  |  |  |  | 16-17 | 8.5 | 9.5 | 7.4 |  |  |  |  |  |  |  | 14-15 | 7.2 |  |  |
| Sri Lanka | 14.8 | 18.4 | 11.3 |  |  |  |  | 13-17 | 15.5 | 19.3 | 11.7 |  |  |  |  |  |  |  |  |  |  |  |
|  |  |  |  |  |  |  |  | 13-15 | 17.2 | 21.3 | 13.2 |  |  |  |  |  |  |  |  |  |  |  |
|  |  |  |  |  |  |  |  | 16-17 | 11.6 | 14.6 | 8.8 |  |  |  |  |  |  |  |  |  |  |  |
| Thailand | 22.5 | 29.8 | 15 | 20%–26% |  |  |  | 13-17 | 11.9 | 18.7 | 5.7 |  |  |  |  | 11.9 |  |  | 12-15 | 11.9 | 17.3 | 6.8 |
|  |  |  |  |  |  |  |  | 13-15 | 12.6 | 17.9 | 7 |  |  |  |  |  |  |  | 12-13 | 12.2 |  |  |
|  |  |  |  |  |  |  |  | 16-17 | 10.3 | 20.9 | 3.5 |  |  |  |  |  |  |  | 14-15 | 11.7 |  |  |
| Timor-Leste | 10.6 | 14.5 | 6.6 |  |  |  |  | 13-17 | 9.8 | 14.5 | 5.3 |  |  |  |  | 8.9 |  |  | 12-15 | 8.5 | 10.9 | 5.8 |
|  |  |  |  |  |  |  |  | 13-15 | 8.4 | 11.3 | 5.6 |  |  |  |  |  |  |  | 12-13 | 8.6 |  |  |
|  |  |  |  |  |  |  |  | 16-17 | 11 | 16.9 | 5.1 |  |  |  |  |  |  |  | 14-15 | 8.1 |  |  |
| Vietnam | 13.7 | 17.7 | 9.4 |  |  |  |  | 13-17 | 19.7 | 26.8 | 13.4 |  |  |  |  | 13.1 |  |  | 12-15 | 13.1 | 17 | 9.5 |
|  |  |  |  |  |  |  |  | 13-15 | 18.2 | 23.7 | 13.2 |  |  |  |  |  |  |  | 14-15 | 13 |  |  |
|  |  |  |  |  |  |  |  | 16-17 | 21.3 | 29.8 | 13.7 |  |  |  |  |  |  |  |  |  |  |  |
|  |  |  |  |  |  |  |  |  |  |  |  |  |  |  |  |  |  |  |  |  |  |  |
| **High-income Asia Pacific** |  |  |  |  |  |  |  |  |  |  |  |  |  |  |  |  |  |  |  |  |  |  |
| Japan |  |  |  |  |  |  |  |  |  |  |  |  |  |  |  |  |  |  |  |  |  |  |
| Singapore | 23.7 | 30.3 | 16.9 |  |  |  |  |  |  |  |  |  |  |  |  |  |  |  |  |  |  |  |
| South Korea | 5.8 | 8.6 | 2.8 | <20% |  |  |  |  |  |  |  |  |  |  |  |  |  |  |  |  |  |  |
|  |  |  |  |  |  |  |  |  |  |  |  |  |  |  |  |  |  |  |  |  |  |  |
| **High-income western countries** |  |  |  |  |  |  |  |  |  |  |  |  |  |  |  |  |  |  |  |  |  |  |
| Australia | 11 | 13.2 | 8.6 | 20%–26% |  |  |  |  |  |  |  | 55.4 | 9-10 | 14 | 6 |  |  |  |  |  |  |  |
| Austria | 22.2 | 28.8 | 15.5 |  | 11 | 31 | 24 |  |  |  |  |  |  |  |  | 19.4 |  |  |  |  |  |  |
|  |  |  |  |  | 13 | 27 | 13 |  |  |  |  |  |  |  |  |  |  |  |  |  |  |  |
|  |  |  |  |  | 15 | 19 | 10 |  |  |  |  |  |  |  |  |  |  |  |  |  |  |  |
| Belgium | 16.5 | 20.8 | 12 |  |  |  |  |  |  |  |  |  |  |  |  | 16.6 |  |  |  |  |  |  |
| Belgium (Flemish) |  |  |  | <20% | 11 | 25 | 20 |  |  |  |  |  |  |  |  |  |  |  |  |  |  |  |
|  |  |  |  |  | 13 | 22 | 12 |  |  |  |  |  |  |  |  |  |  |  |  |  |  |  |
|  |  |  |  |  | 15 | 22 | 10 |  |  |  |  |  |  |  |  |  |  |  |  |  |  |  |
| Belgium (French) |  |  |  |  | 11 | 26 | 16 |  |  |  |  |  |  |  |  |  |  |  |  |  |  |  |
|  |  |  |  |  | 13 | 19 | 14 |  |  |  |  |  |  |  |  |  |  |  |  |  |  |  |
|  |  |  |  |  | 15 | 17 | 9 |  |  |  |  |  |  |  |  |  |  |  |  |  |  |  |
| Canada | 23.7 | 29.5 | 17.6 | 34%–39% | 11 | 37 | 30 |  |  |  |  | 42.6 |  |  |  | 25 |  |  |  |  |  |  |
|  |  |  |  |  | 13 | 32 | 20 |  |  |  |  |  |  |  |  |  |  |  |  |  |  |  |
|  |  |  |  |  | 15 | 28 | 14 |  |  |  |  |  |  |  |  |  |  |  |  |  |  |  |
| Denmark | 15.5 | 17.9 | 13 | 20%–26% | 11 | 13 | 10 |  |  |  |  |  | 9-10 | 12 | 2 | 13.1 |  |  |  |  |  |  |
|  |  |  |  |  | 13 | 12 | 8 |  |  |  |  |  |  |  |  |  |  |  |  |  |  |  |
|  |  |  |  |  | 15 | 13 | 7 |  |  |  |  |  |  |  |  |  |  |  |  |  |  |  |
| Greenland | 26.1 | 31.6 | 20.5 |  | 11 | 26 | 27 |  |  |  |  |  |  |  |  | 18 |  |  |  |  |  |  |
|  |  |  |  |  | 13 | 20 | 14 |  |  |  |  |  |  |  |  |  |  |  |  |  |  |  |
|  |  |  |  |  | 15 | 19 | 13 |  |  |  |  |  |  |  |  |  |  |  |  |  |  |  |
| Finland | 24.6 | 31 | 17.9 | 27%–33% | 11 | 52 | 38 |  |  |  |  | 61.4 |  |  |  | 27.9 |  |  |  |  |  |  |
|  |  |  |  |  | 13 | 33 | 24 |  |  |  |  |  |  |  |  |  |  |  |  |  |  |  |
|  |  |  |  |  | 15 | 22 | 12 |  |  |  |  |  |  |  |  |  |  |  |  |  |  |  |
| France | 13 | 17.6 | 8.2 | 27%–33% | 11 | 17 | 9 |  |  |  |  |  |  |  |  | 12.9 |  |  |  |  |  |  |
|  |  |  |  |  | 13 | 14 | 7 |  |  |  |  |  |  |  |  |  |  |  |  |  |  |  |
|  |  |  |  |  | 15 | 11 | 4 |  |  |  |  |  |  |  |  |  |  |  |  |  |  |  |
| Germany | 16.3 | 20.3 | 12.1 | 27%–33% | 11 | 21 | 14 |  |  |  |  |  |  |  |  | 15.4 |  |  |  |  |  |  |
|  |  |  |  |  | 13 | 16 | 9 |  |  |  |  |  |  |  |  |  |  |  |  |  |  |  |
|  |  |  |  |  | 15 | 13 | 7 |  |  |  |  |  |  |  |  |  |  |  |  |  |  |  |
| Greece | 15.5 | 20 | 10.9 |  | 11 | 24 | 18 |  |  |  |  |  |  |  |  | 13.4 |  |  |  |  |  |  |
|  |  |  |  |  | 13 | 19 | 11 |  |  |  |  |  |  |  |  |  |  |  |  |  |  |  |
|  |  |  |  |  | 15 | 17 | 9 |  |  |  |  |  |  |  |  |  |  |  |  |  |  |  |
| Iceland | 19.7 | 24.6 | 14.8 |  | 11 | 26 | 20 |  |  |  |  |  |  |  |  | 22.4 |  |  |  |  |  |  |
|  |  |  |  |  | 13 | 24 | 15 |  |  |  |  |  |  |  |  |  |  |  |  |  |  |  |
|  |  |  |  |  | 15 | 23 | 16 |  |  |  |  |  |  |  |  |  |  |  |  |  |  |  |
| Ireland | 28.2 | 36.5 | 19.5 |  | 11 | 44 | 33 |  |  |  |  |  |  |  |  | 23.6 |  |  |  |  |  |  |
|  |  |  |  |  | 13 | 29 | 19 |  |  |  |  |  |  |  |  |  |  |  |  |  |  |  |
|  |  |  |  |  | 15 | 20 | 11 |  |  |  |  |  |  |  |  |  |  |  |  |  |  |  |
| Israel | 15.3 | 19.9 | 10.5 |  |  |  |  |  |  |  |  |  |  |  |  | 12.4 |  |  |  |  |  |  |
|  |  |  |  |  |  |  |  |  |  |  |  |  |  |  |  |  |  |  |  |  |  |  |
|  |  |  |  |  |  |  |  |  |  |  |  |  |  |  |  |  |  |  |  |  |  |  |
| Italy | 11.4 | 14.1 | 8.5 |  | 11 | 18 | 7 |  |  |  |  |  |  |  |  | 10.3 |  |  |  |  |  |  |
|  |  |  |  |  | 13 | 11 | 5 |  |  |  |  |  |  |  |  |  |  |  |  |  |  |  |
|  |  |  |  |  | 15 | 7 | 4 |  |  |  |  |  |  |  |  |  |  |  |  |  |  |  |
| Luxembourg | 20.8 | 26.6 | 14.6 |  | 11 | 21 | 14 |  |  |  |  |  |  |  |  | 22.8 |  |  |  |  |  |  |
|  |  |  |  |  | 13 | 18 | 10 |  |  |  |  |  |  |  |  |  |  |  |  |  |  |  |
|  |  |  |  |  | 15 | 17 | 6 |  |  |  |  |  |  |  |  |  |  |  |  |  |  |  |
| Malta | 18.6 | 23.3 | 14.2 |  | 11 | 29 | 19 |  |  |  |  |  |  |  |  | 17.9 |  |  |  |  |  |  |
|  |  |  |  |  | 13 | 22 | 14 |  |  |  |  |  |  |  |  |  |  |  |  |  |  |  |
|  |  |  |  |  | 15 | 15 | 5 |  |  |  |  |  |  |  |  |  |  |  |  |  |  |  |
| Netherlands | 19.8 | 23.4 | 16.1 | 47%–53% | 11 | 23 | 17 |  |  |  |  |  |  |  |  | 18.3 |  |  |  |  |  |  |
|  |  |  |  |  | 13 | 19 | 13 |  |  |  |  |  |  |  |  |  |  |  |  |  |  |  |
|  |  |  |  |  | 15 | 21 | 14 |  |  |  |  |  |  |  |  |  |  |  |  |  |  |  |
| New Zealand | 11.3 | 15.1 | 7.3 | 20%–26% |  |  |  |  |  |  |  |  |  |  |  |  |  |  |  |  |  |  |
| Norway | 16.5 | 21.4 | 11.4 |  | 11 | 22 | 19 |  |  |  |  |  | 9-10 | 30 | 12 | 18.9 |  |  |  |  |  |  |
|  |  |  |  |  | 13 | 15 | 11 |  |  |  |  |  |  |  |  |  |  |  |  |  |  |  |
|  |  |  |  |  | 15 | 13 | 9 |  |  |  |  |  |  |  |  |  |  |  |  |  |  |  |
| Portugal | 15.7 | 21.9 | 9.3 | 27%–33% | 11 | 16 | 9 |  |  |  |  | 35.1 | 9-10 | 16 | 10 | 15.8 |  |  |  |  |  |  |
|  |  |  |  |  | 13 | 14 | 7 |  |  |  |  |  |  |  |  |  |  |  |  |  |  |  |
|  |  |  |  |  | 15 | 12 | 5 |  |  |  |  |  |  |  |  |  |  |  |  |  |  |  |
| Spain | 23.4 | 30.2 | 16.2 | 27%–33% | 11 | 34 | 23 |  |  |  |  |  |  |  |  | 25.9 |  |  |  |  |  |  |
|  |  |  |  |  | 13 | 30 | 14 |  |  |  |  |  |  |  |  |  |  |  |  |  |  |  |
|  |  |  |  |  | 15 | 24 | 9 |  |  |  |  |  |  |  |  |  |  |  |  |  |  |  |
| Sweden | 15.3 | 17.8 | 12.7 | 34%–39% | 11 | 23 | 13 |  |  |  |  |  |  |  |  | 14.1 |  |  |  |  |  |  |
|  |  |  |  |  | 13 | 15 | 14 |  |  |  |  |  |  |  |  |  |  |  |  |  |  |  |
|  |  |  |  |  | 15 | 13 | 9 |  |  |  |  |  |  |  |  |  |  |  |  |  |  |  |
| Switzerland | 14.3 | 17.5 | 10.9 |  | 11 | 23 | 15 |  |  |  |  |  |  |  |  | 14.4 |  |  |  |  |  |  |
|  |  |  |  |  | 13 | 15 | 11 |  |  |  |  |  |  |  |  |  |  |  |  |  |  |  |
|  |  |  |  |  | 15 | 15 | 8 |  |  |  |  |  |  |  |  |  |  |  |  |  |  |  |
| UK | 20.1 | 25.3 | 14.6 |  |  |  |  |  |  |  |  | 50.7 |  |  |  |  |  |  |  |  |  |  |
| England |  |  |  | 40%–46% | 11 | 22 | 18 |  |  |  |  |  | 9-10 | 7 | 1 | 18.5 |  |  |  |  |  |  |
|  |  |  |  |  | 13 | 18 | 9 |  |  |  |  |  | 12-13 | 7 | 2 |  |  |  |  |  |  |  |
|  |  |  |  |  | 15 | 15 | 7 |  |  |  |  |  |  |  |  |  |  |  |  |  |  |  |
| Guernsey Channel Islands |  |  |  | 27%–33% |  |  |  |  |  |  |  |  |  |  |  |  |  |  |  |  |  |  |
| Jersey |  |  |  | 20%–26% |  |  |  |  |  |  |  |  |  |  |  |  |  |  |  |  |  |  |
| Scotland |  |  |  | <20% | 11 | 22 | 20 |  |  |  |  |  |  |  |  | 17.5 |  |  |  |  |  |  |
|  |  |  |  |  | 13 | 19 | 15 |  |  |  |  |  |  |  |  |  |  |  |  |  |  |  |
|  |  |  |  |  | 15 | 16 | 10 |  |  |  |  |  |  |  |  |  |  |  |  |  |  |  |
| Wales |  |  |  | 34%–39% | 11 | 31 | 22 |  |  |  |  |  |  |  |  | 16.3 |  |  |  |  |  |  |
|  |  |  |  |  | 13 | 23 | 13 |  |  |  |  |  |  |  |  |  |  |  |  |  |  |  |
|  |  |  |  |  | 15 | 16 | 7 |  |  |  |  |  |  |  |  |  |  |  |  |  |  |  |
| USA | 28 | 35.6 | 19.5 | 20%–26% |  |  |  |  |  |  |  | 26.5 | 9-10 | 9 | 2 | 32.3 |  |  |  |  |  |  |
|  |  |  |  |  |  |  |  |  |  |  |  |  | 12-13 | 6 | 1 |  |  |  |  |  |  |  |
|  |  |  |  |  |  |  |  |  |  |  |  |  |  |  |  |  |  |  |  |  |  |  |
| **Latin America and Caribbean** |  |  |  |  |  |  |  |  |  |  |  |  |  |  |  |  |  |  |  |  |  |  |
| Anguilla | 18.1 | 21.4 | 14.9 |  |  |  |  | 13-17 | 19.5 | 22 | 17.1 |  |  |  |  |  |  |  |  |  |  |  |
|  |  |  |  |  |  |  |  | 13-15 | 20.3 | 23.1 | 17.4 |  |  |  |  |  |  |  |  |  |  |  |
|  |  |  |  |  |  |  |  | 16-17 | 17.5 | 18.9 | 16.3 |  |  |  |  |  |  |  |  |  |  |  |
| Antigua and Barbuda | 20.8 | 26.1 | 15.4 |  |  |  |  | 13-15 | 31.8 | 36.5 | 27 |  |  |  |  | 22.7 |  |  | 12-15 | 22.4 | 28.4 | 16.4 |
|  |  |  |  |  |  |  |  |  |  |  |  |  |  |  |  |  |  |  | 12-13 | 20.4 |  |  |
|  |  |  |  |  |  |  |  |  |  |  |  |  |  |  |  |  |  |  | 14-15 | 23.6 |  |  |
| Argentina | 15.2 | 20.1 | 10.1 |  |  |  |  | 13-15 | 28.3 | 35.3 | 21.9 |  |  |  |  | 18.2 |  |  | 12-15 | 16.4 | 21.2 | 12.1 |
|  |  |  |  |  |  |  |  |  |  |  |  |  |  |  |  |  |  |  | 12-13 | 17 |  |  |
|  |  |  |  |  |  |  |  |  |  |  |  |  |  |  |  |  |  |  | 14-15 | 16.2 |  |  |
| Bahamas | 15.6 | 19.4 | 11.8 |  |  |  |  | 13-15 | 15.9 | 19.4 | 12.5 |  |  |  |  | 14.6 |  |  | 12-15 | 15.5 | 19.6 | 11.9 |
|  |  |  |  |  |  |  |  |  |  |  |  |  |  |  |  |  |  |  | 12-13 | 15 |  |  |
|  |  |  |  |  |  |  |  |  |  |  |  |  |  |  |  |  |  |  | 14-15 | 16.3 |  |  |
| Barbados | 18.2 | 23.1 | 13.1 |  |  |  |  | 13-15 | 29.1 | 34.5 | 23.3 |  |  |  |  | 18.2 |  |  | 12-15 | 19.4 | 25 | 13.7 |
|  |  |  |  |  |  |  |  |  |  |  |  |  |  |  |  |  |  |  | 12-13 | 17.5 |  |  |
|  |  |  |  |  |  |  |  |  |  |  |  |  |  |  |  |  |  |  | 14-15 | 19.9 |  |  |
| Belize | 19.7 | 23.6 | 15.8 |  |  |  |  | 13-15 | 29 | 32.5 | 25.6 |  |  |  |  | 20.9 |  |  | 12-15 | 20 | 23.6 | 16.7 |
|  |  |  |  |  |  |  |  |  |  |  |  |  |  |  |  |  |  |  | 12-13 | 20.8 |  |  |
|  |  |  |  |  |  |  |  |  |  |  |  |  |  |  |  |  |  |  | 14-15 | 19.4 |  |  |
| Bolivia | 14.5 | 17.6 | 11.4 |  |  |  |  | 13-15 | 24.1 | 29 | 19.8 |  |  |  |  | 13.5 |  |  | 12-15 | 13.4 | 16.5 | 11.2 |
|  |  |  |  |  |  |  |  |  |  |  |  |  |  |  |  |  |  |  | 12-13 | 14 |  |  |
|  |  |  |  |  |  |  |  |  |  |  |  |  |  |  |  |  |  |  | 14-15 | 13.7 |  |  |
| Brazil | 16.4 | 22 | 10.6 | 27%–33% |  |  |  |  |  |  |  | 43.9 | 12-13 | 7 | 2 | 12 |  |  |  |  |  |  |
| British Virgin Islands | 17.8 | 22.5 | 13.1 |  |  |  |  | 13-15 | 27.4 | 33.2 | 22.7 |  |  |  |  | 18 |  |  |  |  |  |  |
| Cayman Islands | 15.2 | 19.4 | 11.1 |  |  |  |  | 13-15 | 16 | 19.8 | 11.7 |  |  |  |  |  |  |  |  |  |  |  |
| Chile | 12.4 | 15.8 | 8.8 | 20%–26% |  |  |  | 13-17 | 13.7 | 19.2 | 8.4 |  |  |  |  | 15.2 |  |  | 12-15 | 15.5 | 22.5 | 8.7 |
|  |  |  |  |  |  |  |  | 13-15 | 14.8 | 21.8 | 7.7 |  |  |  |  |  |  |  | 12-13 | 17.1 |  |  |
|  |  |  |  |  |  |  |  | 16-17 | 12.1 | 15 | 9.6 |  |  |  |  |  |  |  | 14-15 | 14.5 |  |  |
| Colombia | 16.1 | 19 | 13 | 34%–39% |  |  |  |  |  |  |  | 59.5 |  |  |  |  |  |  |  |  |  |  |
| Bogotá |  |  |  |  |  |  |  | 13-15 | 15.1 | 18.1 | 12.8 |  |  |  |  |  |  |  |  |  |  |  |
| Bucaramanga City |  |  |  |  |  |  |  | 13-15 | 16.6 | 19.8 | 13.9 |  |  |  |  |  |  |  |  |  |  |  |
| Cali City |  |  |  |  |  |  |  | 13-15 | 17.1 | 19.7 | 15 |  |  |  |  |  |  |  |  |  |  |  |
| Manizales |  |  |  |  |  |  |  | 13-15 | 12.1 | 16 | 9.1 |  |  |  |  |  |  |  |  |  |  |  |
| Valledupar City |  |  |  |  |  |  |  | 13-15 | 14.3 | 17.8 | 11.7 |  |  |  |  |  |  |  |  |  |  |  |
| Costa Rica | 18 | 23.9 | 11.8 |  |  |  |  | 13-15 | 27.4 | 35.9 | 19 |  |  |  |  | 18.1 |  |  | 12-15 | 18 | 24.7 | 11.4 |
|  |  |  |  |  |  |  |  |  |  |  |  |  |  |  |  |  |  |  | 12-13 | 19 |  |  |
|  |  |  |  |  |  |  |  |  |  |  |  |  |  |  |  |  |  |  | 14-15 | 17.5 |  |  |
| Curaçao |  |  |  |  |  |  |  | 13-17 | 11.4 | 15.2 | 7.8 |  |  |  |  | 11.7 |  |  | 12-15 | 11.6 | 15.5 | 7.9 |
|  |  |  |  |  |  |  |  | 13-15 | 11.5 | 15.8 | 7.3 |  |  |  |  |  |  |  | 12-13 | 11.5 |  |  |
|  |  |  |  |  |  |  |  | 16-17 | 11.2 | 14.3 | 8.4 |  |  |  |  |  |  |  | 14-15 | 11.6 |  |  |
| Dominica | 15.9 | 17.9 | 14 |  |  |  |  | 13-15 | 23.7 | 24.8 | 22.6 |  |  |  |  | 16.1 |  |  |  |  |  |  |
| Dominican Republic |  |  |  |  |  |  |  | 13-17 | 12.5 | 15.2 | 10.4 |  |  |  |  |  |  |  |  |  |  |  |
|  |  |  |  |  |  |  |  | 13-15 | 14.7 | 17.7 | 12 |  |  |  |  |  |  |  |  |  |  |  |
|  |  |  |  |  |  |  |  | 16-17 | 9.9 | 12.4 | 8.1 |  |  |  |  |  |  |  |  |  |  |  |
| Ecuador | 13.5 | 16.8 | 10 | 27%–33% |  |  |  |  |  |  |  |  |  |  |  |  |  |  |  |  |  |  |
| Guayaquil |  |  |  |  |  |  |  | 13-15 | 8.7 | 12.2 | 5.8 |  |  |  |  |  |  |  |  |  |  |  |
| Quito |  |  |  |  |  |  |  | 13-15 | 14.8 | 18.9 | 10.8 |  |  |  |  |  |  |  |  |  |  |  |
| Zamora |  |  |  |  |  |  |  | 13-15 | 14.9 | 17.5 | 12 |  |  |  |  |  |  |  |  |  |  |  |
| El Salvador | 13.9 | 17.2 | 10.5 |  |  |  |  | 13-15 | 12.7 | 15.9 | 9.5 |  |  |  |  | 13.8 |  |  | 12-15 | 12.5 | 15.9 | 9.1 |
|  |  |  |  |  |  |  |  |  |  |  |  |  |  |  |  |  |  |  | 12-13 | 8.7 |  |  |
|  |  |  |  |  |  |  |  |  |  |  |  |  |  |  |  |  |  |  | 14-15 | 14.2 |  |  |
| Grenada | 15.7 | 18.2 | 13.1 |  |  |  |  | 13-15 | 15.2 | 18.1 | 13.1 |  |  |  |  |  |  |  |  |  |  |  |
| Guatemala | 13.1 | 15.5 | 10.6 |  |  |  |  | 13-17 | 10.5 | 12.2 | 8.4 |  |  |  |  | 14 |  |  | 12-15 | 11.1 | 12.8 | 9.7 |
|  |  |  |  |  |  |  |  | 13-15 | 10.9 | 12.7 | 8.7 |  |  |  |  |  |  |  | 12-13 | 12.3 |  |  |
|  |  |  |  |  |  |  |  | 16-17 | 8.6 | 10.2 | 6.5 |  |  |  |  |  |  |  | 14-15 | 10.8 |  |  |
| Guyana | 16 | 18.3 | 13.7 |  |  |  |  | 11-18 | 21.3 | 23.8 | 19.1 |  |  |  |  | 15.6 |  |  | 12-15 | 14.9 | 18.4 | 12.1 |
|  |  |  |  |  |  |  |  |  |  |  |  |  |  |  |  |  |  |  | 12-13 | 13.6 |  |  |
|  |  |  |  |  |  |  |  |  |  |  |  |  |  |  |  |  |  |  | 14-15 | 15.6 |  |  |
| Honduras | 16.2 | 19.8 | 12.4 |  |  |  |  | 13-15 | 20.8 | 24.7 | 17.5 |  |  |  |  | 15.7 |  |  | 12-15 | 15.1 | 18.1 | 12.6 |
| Honduras |  |  |  |  |  |  |  |  |  |  |  |  |  |  |  |  |  |  | 12-13 | 14.6 |  |  |
|  |  |  |  |  |  |  |  |  |  |  |  |  |  |  |  |  |  |  | 14-15 | 15.8 |  |  |
| Jamaica |  |  |  |  |  |  |  | 13-17 | 23.2 | 23.8 | 22.6 |  |  |  |  |  |  |  |  |  |  |  |
|  |  |  |  |  |  |  |  | 13-15 | 23.8 | 25.4 | 22.2 |  |  |  |  |  |  |  |  |  |  |  |
|  |  |  |  |  |  |  |  | 16-17 | 22.4 | 21.9 | 23.1 |  |  |  |  |  |  |  |  |  |  |  |
| Mexico | 16.8 | 21.2 | 12.4 | 34%–39% |  |  |  |  |  |  |  |  |  |  |  | 17.8 |  |  |  |  |  |  |
| Montserrat | 20.1 | 20.9 | 19.2 |  |  |  |  | 13-15 | 22.9 | 22.1 | 23.6 |  |  |  |  |  |  |  |  |  |  |  |
| Netherlands Antilles | 13.1 | 16.5 | 9.7 |  |  |  |  |  |  |  |  |  |  |  |  |  |  |  |  |  |  |  |
| Paraguay | 16.5 | 21 | 11.8 |  |  |  |  | 13-17 | 17.2 | 22.4 | 12.1 |  |  |  |  |  |  |  |  |  |  |  |
|  |  |  |  |  |  |  |  | 13-15 | 17.4 | 23 | 12.2 |  |  |  |  |  |  |  |  |  |  |  |
|  |  |  |  |  |  |  |  | 16-17 | 16.9 | 21.5 | 11.9 |  |  |  |  |  |  |  |  |  |  |  |
| Peru | 15.3 | 17.3 | 13.2 |  |  |  |  | 13-15 | 24.5 | 27 | 22.2 |  |  |  |  | 15.4 |  |  | 12-15 | 15.2 | 17.1 | 13.3 |
|  |  |  |  |  |  |  |  |  |  |  |  |  |  |  |  |  |  |  | 12-13 | 18.1 |  |  |
|  |  |  |  |  |  |  |  |  |  |  |  |  |  |  |  |  |  |  | 14-15 | 14.1 |  |  |
| Saint Kitts and Nevis | 17.7 | 21.5 | 13.9 |  |  |  |  | 13-15 | 25.6 | 29.3 | 21.9 |  |  |  |  | 18 |  |  |  |  |  |  |
| Saint Lucia | 15.7 | 17.2 | 14.1 |  |  |  |  | 13-15 | 15.6 | 17.4 | 14 |  |  |  |  |  |  |  |  |  |  |  |
| Saint Vincent and the Grenadines | 14.2 | 16.8 | 11.5 |  |  |  |  | 13-15 | 13.3 | 15.6 | 11.4 |  |  |  |  |  |  |  |  |  |  |  |
| Suriname | 18.6 | 21.6 | 15.4 |  |  |  |  | 13-17 | 19.3 | 21.7 | 16.8 |  |  |  |  | 19.2 |  |  | 12-15 | 20.2 | 24.8 | 16.2 |
|  |  |  |  |  |  |  |  | 13-15 | 18.8 | 21.2 | 16.6 |  |  |  |  |  |  |  | 12-13 | 21.2 |  |  |
|  |  |  |  |  |  |  |  | 16-17 | 20.8 | 22.8 | 17.7 |  |  |  |  |  |  |  | 14-15 | 19.6 |  |  |
| Trinidad and Tobago | 17.9 | 21.2 | 14.4 |  |  |  |  | 13-17 | 20.1 | 25.5 | 15 |  |  |  |  | 19.9 |  |  | 12-15 | 20.2 | 25 | 15.8 |
|  |  |  |  |  |  |  |  | 13-15 | 20.5 | 24.9 | 16.3 |  |  |  |  |  |  |  | 12-13 | 20.7 |  |  |
|  |  |  |  |  |  |  |  | 16-17 | 19.3 | 27.1 | 12.2 |  |  |  |  |  |  |  | 14-15 | 20 |  |  |
| Uruguay | 17.8 | 24.7 | 10.6 | 27%–33% |  |  |  | 13-15 | 28.8 | 42.6 | 17.1 |  |  |  |  | 15.2 |  |  | 12-15 | 16.1 | 23.6 | 9.6 |
|  |  |  |  |  |  |  |  |  |  |  |  |  |  |  |  |  |  |  | 12-13 | 16.3 |  |  |
|  |  |  |  |  |  |  |  |  |  |  |  |  |  |  |  |  |  |  | 14-15 | 16 |  |  |
| Venezuela | 11.2 | 15.2 | 7.1 | 27%–33% |  |  |  |  |  |  |  |  |  |  |  |  |  |  |  |  |  |  |
| Barinas |  |  |  |  |  |  |  | 13-15 | 8.1 | 11.8 | 4.8 |  |  |  |  |  |  |  |  |  |  |  |
| Lara |  |  |  |  |  |  |  | 13-15 | 9.4 | 14 | 5.1 |  |  |  |  |  |  |  |  |  |  |  |
|  |  |  |  |  |  |  |  |  |  |  |  |  |  |  |  |  |  |  |  |  |  |  |
| **Oceania** |  |  |  |  |  |  |  |  |  |  |  |  |  |  |  |  |  |  |  |  |  |  |
| American Samoa | 14.2 | 16.3 | 12.2 |  |  |  |  |  |  |  |  |  |  |  |  |  |  |  |  |  |  |  |
| Cook Islands | 17.3 | 22.3 | 12.3 |  |  |  |  | 13-17 | 15.7 | 21.3 | 9.6 |  |  |  |  |  |  |  |  |  |  |  |
|  |  |  |  |  |  |  |  | 13-15 | 16.6 | 20.7 | 11.4 |  |  |  |  |  |  |  |  |  |  |  |
|  |  |  |  |  |  |  |  | 16-17 | 14.5 | 22.1 | 7.2 |  |  |  |  |  |  |  |  |  |  |  |
| Fiji | 16.7 | 19.3 | 14 |  |  |  |  | 13-17 | 20.4 | 22.4 | 18.5 |  |  |  |  | 18.5 |  |  |  |  |  |  |
|  |  |  |  |  |  |  |  | 13-15 | 19.2 | 21.2 | 17.5 |  |  |  |  |  |  |  |  |  |  |  |
|  |  |  |  |  |  |  |  | 16-17 | 21.5 | 23.6 | 19.5 |  |  |  |  |  |  |  |  |  |  |  |
| French Polynesia | 17.2 | 21.8 | 12.5 |  |  |  |  | 13-17 | 18.2 | 23.9 | 12.4 |  |  |  |  |  |  |  |  |  |  |  |
|  |  |  |  |  |  |  |  | 13-15 | 17.3 | 21.3 | 13.4 |  |  |  |  |  |  |  |  |  |  |  |
|  |  |  |  |  |  |  |  | 16-17 | 19.9 | 29.1 | 10.3 |  |  |  |  |  |  |  |  |  |  |  |
| Guam | 21.1 | 25.5 | 16.4 |  |  |  |  |  |  |  |  |  |  |  |  |  |  |  |  |  |  |  |
| Kiribati | 17.5 | 20.7 | 14.1 |  |  |  |  | 13-15 | 26.8 | 32.8 | 21.9 |  |  |  |  | 17.8 |  |  | 12-15 | 17.1 | 20.7 | 14.2 |
|  |  |  |  |  |  |  |  |  |  |  |  |  |  |  |  |  |  |  | 12-13 | 14.6 |  |  |
|  |  |  |  |  |  |  |  |  |  |  |  |  |  |  |  |  |  |  | 14-15 | 18.1 |  |  |
| Nauru | 13.2 | 16.4 | 10 |  |  |  |  | 13-15 | 14.7 | 17.5 | 12.5 |  |  |  |  | 12.6 |  |  |  |  |  |  |
| Niue | 12.7 | 13.9 | 11.5 |  |  |  |  | 13-15 | 31.5 | 41.4 |  |  |  |  |  | 12.4 |  |  |  |  |  |  |
| Palau | 21 | 24.1 | 17.9 |  |  |  |  |  |  |  |  |  |  |  |  |  |  |  |  |  |  |  |
| Samoa | 12.9 | 12.5 | 13.3 |  |  |  |  | 13-15 | 21 | 19.8 | 22.1 |  |  |  |  | 11.8 |  |  | 12-15 | 12.2 | 10.9 | 13.5 |
|  |  |  |  |  |  |  |  |  |  |  |  |  |  |  |  |  |  |  | 12-13 | 10.5 |  |  |
|  |  |  |  |  |  |  |  |  |  |  |  |  |  |  |  |  |  |  | 14-15 | 12.7 |  |  |
| Solomon Islands | 16.3 | 17.9 | 14.6 |  |  |  |  | 13-15 | 27.2 | 30 | 25.1 |  |  |  |  | 16.4 |  |  | 12-15 | 16.2 | 18.4 | 14.5 |
|  |  |  |  |  |  |  |  |  |  |  |  |  |  |  |  |  |  |  | 12-13 | 12.7 |  |  |
|  |  |  |  |  |  |  |  |  |  |  |  |  |  |  |  |  |  |  | 14-15 | 17.6 |  |  |
| Tokelau | 22.8 | 25.9 | 19.7 |  |  |  |  | 13-17 | 23.7 | 27.1 | 20.9 |  |  |  |  | 24 |  |  |  |  |  |  |
|  |  |  |  |  |  |  |  | 13-15 | 23.9 | 20.6 | 29 |  |  |  |  |  |  |  |  |  |  |  |
|  |  |  |  |  |  |  |  | 16-17 | 23.1 |  |  |  |  |  |  |  |  |  |  |  |  |  |
| Tonga | 14.2 | 13.3 | 15.2 |  |  |  |  | 13-17 | 18.3 | 19.5 | 17.1 |  |  |  |  | 13.8 |  |  | 12-15 | 13.8 | 12 | 15.5 |
|  |  |  |  |  |  |  |  | 13-15 | 19.4 | 19.6 | 19.5 |  |  |  |  |  |  |  | 12-13 | 13.5 |  |  |
|  |  |  |  |  |  |  |  | 16-17 | 16 | 19.2 | 12.4 |  |  |  |  |  |  |  | 14-15 | 13.9 |  |  |
| Tuvalu | 12.9 | 14.7 | 11.1 |  |  |  |  | 13-15 | 10.6 | 13 | 8.3 |  |  |  |  | 11.6 |  |  |  |  |  |  |
| Vanuatu | 12.5 | 13.8 | 11 |  |  |  |  | 13-17 | 12.5 | 12 | 13.1 |  |  |  |  | 11 |  |  | 12-15 | 10.7 | 12.4 | 9.4 |
|  |  |  |  |  |  |  |  | 13-15 | 12.4 | 11.8 | 13 |  |  |  |  |  |  |  | 12-13 | 7.4 |  |  |
|  |  |  |  |  |  |  |  | 16-17 | 12.6 | 12.2 | 13.2 |  |  |  |  |  |  |  | 14-15 | 14.2 |  |  |
| Wallis and Futuna | 14.3 | 15.4 | 13.2 |  |  |  |  | 13-17 | 12.8 | 14.9 | 11.1 |  |  |  |  | 13.3 |  |  | 12-15 | 14.4 | 14 | 14.7 |
|  |  |  |  |  |  |  |  | 13-15 | 13.6 | 13.7 | 13.6 |  |  |  |  |  |  |  | 12-13 | 15.5 |  |  |
|  |  |  |  |  |  |  |  | 16-17 | 11.6 | 16.7 | 7.2 |  |  |  |  |  |  |  | 14-15 | 13.9 |  |  |
|  |  |  |  |  |  |  |  |  |  |  |  |  |  |  |  |  |  |  |  |  |  |  |
| **South Asia** |  |  |  |  |  |  |  |  |  |  |  |  |  |  |  |  |  |  |  |  |  |  |
| Afghanistan | 11.9 | 11.5 | 12.2 |  |  |  |  | 13-17 | 9.3 | 9.1 | 9.9 |  |  |  |  | 10.1 |  |  | 12-15 | 8.8 | 7.5 | 11.1 |
|  |  |  |  |  |  |  |  | 13-15 | 9.6 | 9.4 | 10.2 |  |  |  |  |  |  |  | 12-13 | 9.5 |  |  |
|  |  |  |  |  |  |  |  | 16-17 | 8.8 | 8.6 | 9.3 |  |  |  |  |  |  |  | 14-15 | 8.9 |  |  |
| Bangladesh | 33.9 | 36.8 | 30.8 | 40%–46% |  |  |  | 13-17 | 41.4 | 42 | 40.2 |  |  |  |  | 48.2 |  |  | 12-15 | 42.4 | 42.4 | 41.9 |
|  |  |  |  |  |  |  |  | 13-15 | 41.2 | 41.6 | 40.7 |  |  |  |  |  |  |  | 12-13 | 34 | 40.1 |  |
|  |  |  |  |  |  |  |  | 16-17 | 43 | 45.4 |  |  |  |  |  |  |  |  | 14-15 | 45.7 |  |  |
| Bhutan | 15.9 | 17.2 | 14.6 |  |  |  |  | 13-17 | 14.4 | 15.8 | 13.2 |  |  |  |  |  |  |  |  |  |  |  |
|  |  |  |  |  |  |  |  | 13-15 | 15.2 | 16.7 | 13.9 |  |  |  |  |  |  |  |  |  |  |  |
|  |  |  |  |  |  |  |  | 16-17 | 13.5 | 14.8 | 12.4 |  |  |  |  |  |  |  |  |  |  |  |
| India | 26.1 | 28.2 | 23.7 | 27%–33% |  |  |  | 13-15 | 30.2 | 31 | 29.1 | 25 |  |  |  |  |  |  |  |  |  |  |
| Nepal | 16.5 | 18.2 | 14.7 | 34%–39% |  |  |  | 13-17 | 15.2 | 17.4 | 13.4 |  |  |  |  |  |  |  |  |  |  |  |
|  |  |  |  |  |  |  |  | 13-15 | 14.3 | 15.8 | 13.3 |  |  |  |  |  |  |  |  |  |  |  |
|  |  |  |  |  |  |  |  | 16-17 | 17.9 | 21.8 | 13.7 |  |  |  |  |  |  |  |  |  |  |  |
| Pakistan | 13 | 14.6 | 11.4 |  |  |  |  | 13-15 | 15.5 | 17.2 | 12.7 |  |  |  |  | 11.7 |  |  | 12-15 | 11.6 | 12.9 | 9.4 |
|  |  |  |  |  |  |  |  |  |  |  |  |  |  |  |  |  |  |  | 12-13 | 11.9 |  |  |
|  |  |  |  |  |  |  |  |  |  |  |  |  |  |  |  |  |  |  | 14-15 | 11.4 |  |  |
|  |  |  |  |  |  |  |  |  |  |  |  |  |  |  |  |  |  |  |  |  |  |  |
| **Sub-Saharan Africa** |  |  |  |  |  |  |  |  |  |  |  |  |  |  |  |  |  |  |  |  |  |  |
| Benin | 24.1 | 28.7 | 19.3 |  |  |  |  | 13-17 | 29.3 | 32.8 | 21.8 |  |  |  |  |  |  |  | 12-15 | 24.4 | 27.3 | 19.3 |
|  |  |  |  |  |  |  |  | 13-15 | 28.3 | 32.9 | 20.2 |  |  |  |  |  |  |  | 12-13 | 26.4 |  |  |
|  |  |  |  |  |  |  |  | 16-17 | 30 | 32.8 | 23.2 |  |  |  |  |  |  |  | 14-15 | 23.9 |  |  |
| Botswana | 12.5 | 13.9 | 11.1 |  |  |  |  | 13-15 | 11.7 | 13 | 10.4 |  |  |  |  |  |  |  |  |  |  |  |
| Djibouti | 14.8 | 18.7 | 10.7 |  |  |  |  | 13-15 | 14.9 | 18.8 | 9.2 |  |  |  |  |  |  |  |  |  |  |  |
| Ethiopia |  |  |  | 27%–33% |  |  |  |  |  |  |  |  |  |  |  |  |  |  |  |  |  |  |
| Ghana | 12.5 | 13.4 | 11.6 | 47%–53% |  |  |  |  |  |  |  |  |  |  |  | 13 |  |  |  |  |  |  |
| Senior High |  |  |  |  |  |  |  | 13-17 | 23.9 | 26.9 | 21.1 |  |  |  |  |  |  |  |  |  |  |  |
|  |  |  |  |  |  |  |  | 13-15 | 33.4 |  | 33.4 |  |  |  |  |  |  |  |  |  |  |  |
|  |  |  |  |  |  |  |  | 16-17 | 21.2 | 25.5 | 16.8 |  |  |  |  |  |  |  |  |  |  |  |
| Junior High |  |  |  |  |  |  |  | 13-17 | 17.1 | 17.4 | 16.9 |  |  |  |  |  |  |  |  |  |  |  |
|  |  |  |  |  |  |  |  | 13-15 | 16 | 15.9 | 16.1 |  |  |  |  |  |  |  |  |  |  |  |
|  |  |  |  |  |  |  |  | 16-17 | 19.9 | 20.8 | 18.9 |  |  |  |  |  |  |  |  |  |  |  |
| Kenya | 13.2 | 15.1 | 11.1 |  |  |  |  | 13-15 | 12 | 14.2 | 10.1 | 58.1 |  |  |  |  |  |  |  |  |  |  |
| Liberia |  |  |  |  |  |  |  | 13-17 | 10.7 | 12.9 | 8.5 |  |  |  |  |  |  |  |  |  |  |  |
|  |  |  |  |  |  |  |  | 13-15 | 9.2 | 9.9 | 8.3 |  |  |  |  |  |  |  |  |  |  |  |
|  |  |  |  |  |  |  |  | 16-17 | 11.9 | 15 | 8.6 |  |  |  |  |  |  |  |  |  |  |  |
| Mauritania | 12.8 | 16.8 | 8.6 |  |  |  |  | 13-15 | 16.3 | 21.5 | 10.9 |  |  |  |  | 12.1 |  |  | 12-15 | 10.5 | 13.9 | 6.8 |
|  |  |  |  |  |  |  |  |  |  |  |  |  |  |  |  |  |  |  | 12-13 | 9.3 |  |  |
|  |  |  |  |  |  |  |  |  |  |  |  |  |  |  |  |  |  |  | 14-15 | 11.1 |  |  |
| Mauritius | 17.8 | 23.8 | 11.6 |  |  |  |  | 13-17 | 19.1 | 26.5 | 12.5 |  |  |  |  | 19.2 |  |  | 12-15 | 19.4 | 26 | 13.3 |
|  |  |  |  |  |  |  |  | 13-15 | 19.9 | 28 | 13.2 |  |  |  |  |  |  |  | 12-13 | 18.1 |  |  |
|  |  |  |  |  |  |  |  | 16-17 | 17.6 | 24.2 | 11.4 |  |  |  |  |  |  |  | 14-15 | 20.2 |  |  |
| Mozambique | 12.9 | 17 | 8.7 |  |  |  |  | 13-17 | 13.3 | 17.6 | 8.2 | 89 |  |  |  | 13.4 |  |  | 12-15 | 11 | 12.6 | 8.7 |
|  |  |  |  |  |  |  |  | 13-15 | 11.5 | 13.8 | 8.4 |  |  |  |  |  |  |  | 12-13 | 13.7 |  |  |
|  |  |  |  |  |  |  |  | 16-17 | 15.8 | 22.6 | 7.8 |  |  |  |  |  |  |  | 14-15 | 10.1 |  |  |
| Namibia | 12.6 | 13.5 | 11.6 |  |  |  |  | 13-17 | 14.1 | 14.9 | 13.2 |  |  |  |  | 14.1 |  |  | 12-15 | 13.8 | 14.5 | 13.3 |
|  |  |  |  |  |  |  |  | 13-15 | 14.1 | 14.7 | 13.6 |  |  |  |  |  |  |  | 12-13 | 13.8 |  |  |
|  |  |  |  |  |  |  |  | 16-17 | 14.1 | 15.3 | 12.7 |  |  |  |  |  |  |  | 14-15 | 13.7 |  |  |
| Nigeria |  |  |  | 47%–53% |  |  |  |  |  |  |  |  |  |  |  |  |  |  |  |  |  |  |
| Senegal | 11.5 | 15.2 | 7.8 |  |  |  |  | 13-15 | 11.8 | 15.4 | 6.2 |  |  |  |  |  |  |  |  |  |  |  |
| Seychelles | 17.4 | 21.3 | 13.3 |  |  |  |  | 13-17 | 18.2 | 21.9 | 14.7 |  |  |  |  | 16.9 |  |  | 12-15 | 17.5 | 19.8 | 15.4 |
|  |  |  |  |  |  |  |  | 13-15 | 18.3 | 21.6 | 15.4 |  |  |  |  |  |  |  | 12-13 | 16.1 |  |  |
|  |  |  |  |  |  |  |  | 16-17 | 17.8 | 22.7 | 12 |  |  |  |  |  |  |  | 14-15 | 18.7 |  |  |
| South Africa |  |  |  | 47%–53% |  |  |  |  |  |  |  | 51.7 |  |  |  |  |  |  |  |  |  |  |
| Sudan | 9.7 | 10.4 | 9 |  |  |  |  | 13-15 | 11 | 10.9 | 11.2 |  |  |  |  | 8.8 |  |  | 12-15 | 7.8 | 8.2 | 7.4 |
|  |  |  |  |  |  |  |  |  |  |  |  |  |  |  |  |  |  |  | 12-13 | 8.9 |  |  |
|  |  |  |  |  |  |  |  |  |  |  |  |  |  |  |  |  |  |  | 14-15 | 7.3 |  |  |
| Tanzania | 17.9 | 21.8 | 14 |  |  |  |  | <12-18+ | 20 | 23 | 17.2 |  |  |  |  | 20.2 |  |  | 12-15 | 20.7 | 23.6 | 18.4 |
|  |  |  |  |  |  |  |  |  |  |  |  |  |  |  |  |  |  |  | 12-13 | 19.8 |  |  |
|  |  |  |  |  |  |  |  |  |  |  |  |  |  |  |  |  |  |  | 14-15 | 21.3 |  |  |
| Uganda | 14.3 | 16 | 12.7 |  |  |  |  | 13-15 | 15.3 | 16.1 | 14.4 |  |  |  |  |  |  |  |  |  |  |  |
| Zambia | 10.7 | 10.6 | 10.9 |  |  |  |  | 13-15 | 9.8 | 9.7 | 10.2 |  |  |  |  |  |  |  |  |  |  |  |
| Zimbabwe | 13.4 | 15.4 | 11.4 | 54%–59% |  |  |  |  |  |  |  |  |  |  |  |  |  |  |  |  |  |  |
| Bulawayo |  |  |  |  |  |  |  | 13-15 | 12.8 | 14.4 | 11.6 |  |  |  |  |  |  |  |  |  |  |  |
| Harare |  |  |  |  |  |  |  | 13-15 | 14.4 | 16.1 | 12.9 |  |  |  |  |  |  |  |  |  |  |  |
| Manicaland |  |  |  |  |  |  |  | 13-15 | 11.7 | 13 | 10.5 |  |  |  |  |  |  |  |  |  |  |  |

**Notes:** ^a^ = 2016 estimated prevalence of physical activity of 11-17 years-old (%); ^b^ = Estimated prevalence of physical activity of 5-17 years-old; ^c^ = Estimated prevalence of physical activity (%); ^d^ = Estimated prevalence of physical activity of 9-11 years-old (%); ^e^ = Estimated prevalence of physical activity of 11-17 years-old (%); HBSC = Health Behaviour in School-aged Children; GSHS = Global School-Based Student Health Survey; ISCOLE = International Study of Childhood Obesity, Lifestyle and the Environment; ICAD = International Children's Accelerometry Database.
