# Supplementary material for: Global prevalence of physical activity for children and adolescents; inconsistencies, research gaps, and recommendations: a narrative review
Source: Int J Behav Nutr Phys Act. 2021 Jun 29;18:81. doi: 10.1186/s12966-021-01155-2 (PMC8243483; doi:10.1186/s12966-021-01155-2)
Supplement: Supplementary file 3 — Additional file 3. Summary of the characteristics of the datasets by world region for each included initiative [file 12966_2021_1155_MOESM3_ESM.docx]

**Online Supplement Material 3:** Summary of the characteristics of the datasets by world region for each included initiative.

| **Guthold et al. (2020)** |  |  |  |  |  |  |  |  |  |  |  |  |  |  |  | |  |  |  |  |
| --- | --- | --- | --- | --- | --- | --- | --- | --- | --- | --- | --- | --- | --- | --- | --- | --- | --- | --- | --- | --- |
| **WHO area** | **Number of countries with data** | **Survey/study used** | **n (%)** | **Age ranges** | **n (%)** | **Year of data collection** | |  | **Sample size** |  |  |  | **Prevalence of physical activity (%)** | |  |  | |  |  |  |
|  |  |  |  |  |  | **Average** | **Most recent** | **Least recent** | **Average** | **Median** | **Max** | **Min** | **Average** | **Max** | **Country with Max** | **Min** | | **Country with Min** | **Male Average** | **Female Average** |
| **Central and eastern Europe** | 16 | HBSC | 16 (100) | 11-15 | 16 (100) | 2014 | 2014 | 2014 | 4820 | 4723 | 6016 | 3876 | 19.1 | 28.5 | Slovakia | 15.5 | | Russia | 23.8 | 14.1 |
| **Central Asia, Middle East, and North Africa** | 19 | GSHS | 17 (89) | 11-16 | 9 (47) | 2012 | 2016 | 2007 | 3726 | 3090 | 7017 | 1829 | 15 | 22.3 | Armenia | 11.8 | | Qatar | 19.8 | 10.1 |
|  |  | HBSC | 2 (11) | 11-17 | 8 (42) |  |  |  |  |  |  |  |  |  |  |  | |  |  |  |
|  |  |  |  | 11-15 | 2 (11) |  |  |  |  |  |  |  |  |  |  |  | |  |  |  |
|  |  |  |  | 12-17 | 1 (5) |  |  |  |  |  |  |  |  |  |  |  | |  |  |  |
| **East and Southeast Asia** | 14 | GSHS | 13 (93) | 11-16 | 2 (14) | 2013 | 2016 | 2003 | 6111 | 3115 | 25981 | 1160 | 14.5 | 22.5 | Thailand | 6.6 | | Philippines | 18 | 10.7 |
|  |  | *Physical Activity Surveillance System* | 1 (7) | 11-17 | 6 (43) |  |  |  |  |  |  |  |  |  |  |  | |  |  |  |
|  |  |  |  | 12-17 | 3 (21) |  |  |  |  |  |  |  |  |  |  |  | |  |  |  |
|  |  |  |  | 13-17 | 2 (14) |  |  |  |  |  |  |  |  |  |  |  | |  |  |  |
|  |  |  |  | 12-16 | 1 (7) |  |  |  |  |  |  |  |  |  |  |  | |  |  |  |
| **High-income Asia Pacific** | 2 | *Students' Health Survey* | 1 (50) | 13-17 | 2 (100) | 2014 | 2015 | 2013 | 34787 | 34787 | 60423 | 9151 | 7.8 | 23.7 | Singapore | 5.8 | | South Korea | 11 | 4.4 |
|  |  | KYRBS | 1 (50) |  |  |  |  |  |  |  |  |  |  |  |  |  | |  |  |  |
| **High-income western countries** | 26 | HBSC | 23 (88) | 11-15 | 23 (88) | 2014 | 2015 | 2010 | 6453 | 4990 | 16244 | 1205 | 21.8 | 28.2 | Ireland | 11 | | Australia | 27.9 | 15.4 |
|  |  | NNPAS | 1 (4) | 11-17 | 1 (4) |  |  |  |  |  |  |  |  |  |  |  | |  |  |  |
|  |  | *The Youth 12 survey* | 1 (4) | 13-17 | 1 (4) |  |  |  |  |  |  |  |  |  |  |  | |  |  |  |
|  |  | YRBSS | 1 (4) | 14-17 | 1 (4) |  |  |  |  |  |  |  |  |  |  |  | |  |  |  |
| **Latin America and Caribbean** | 32 | GSHS | 30 (94) | 11-16 | 12 (38) | 2011 | 2016 | 2003 | 3539 | 1856 | 27034 | 197 | 15.7 | 26.1 | Antigua and Barbuda | 15.2 | | Venezuela | 20.1 | 11.1 |
|  |  | PeNSE | 1 (3) | 12-16 | 10 (31) |  |  |  |  |  |  |  |  |  |  |  | |  |  |  |
|  |  | ENSANUT | 1 (3) | 12-17 | 3 (9) |  |  |  |  |  |  |  |  |  |  |  | |  |  |  |
|  |  |  |  | 13-17 | 1 (3) |  |  |  |  |  |  |  |  |  |  |  | |  |  |  |
|  |  |  |  | 13-16 | 3 (9) |  |  |  |  |  |  |  |  |  |  |  | |  |  |  |
|  |  |  |  | 11-14 | 1 (3) |  |  |  |  |  |  |  |  |  |  |  | |  |  |  |
|  |  |  |  | 11-17 | 2 (6) |  |  |  |  |  |  |  |  |  |  |  | |  |  |  |
| **Oceania** | 16 | GSHS | 13 (81) | 11-15 | 1 (6) | 2012 | 2016 | 2010 | 1345 | 1190 | 2916 | 66 | 16 | 25.9 | Tokelau | 12.5 | | Samoa | 18.1 | 13.8 |
|  |  | YRBS | 3 (19) | 11-16 | 3 (19) |  |  |  |  |  |  |  |  |  |  |  | |  |  |  |
|  |  |  |  | 11-17 | 1 (6) |  |  |  |  |  |  |  |  |  |  |  | |  |  |  |
|  |  |  |  | 12-16 | 5 (31) |  |  |  |  |  |  |  |  |  |  |  | |  |  |  |
|  |  |  |  | 12-17 | 1 (6) |  |  |  |  |  |  |  |  |  |  |  | |  |  |  |
|  |  |  |  | 13-17 | 2 (12) |  |  |  |  |  |  |  |  |  |  |  | |  |  |  |
|  |  |  |  | 14-17 | 2 (12) |  |  |  |  |  |  |  |  |  |  |  | |  |  |  |
|  |  |  |  | 15-17 | 1 (6) |  |  |  |  |  |  |  |  |  |  |  | |  |  |  |
| **South Asia** | 6 | GSHS | 6 (100) | 11-17 | 3 (50) | 2013 | 2016 | 2155 | 5017 | 5577.5 | 7744 | 2155 | 24.8 | 33.9 | Bangladesh | 11.9 | | Afghanistan | 26.9 | 22.5 |
|  |  |  |  | 11-16 | 2 (33) |  |  |  |  |  |  |  |  |  |  |  | |  |  |  |
|  |  |  |  | 12-17 | 1 (14) |  |  |  |  |  |  |  |  |  |  |  | |  |  |  |
| **Sub-Saharan Africa** | 16 | GSHS | 16 (100) | 11-16 | 10 (62) | 2009 | 2015 | 2003 | 2825 | 2409 | 5925 | 1325 | 13.8 | 24.1 | Benin | 9.7 | | Sudan | 16.1 | 11.5 |
|  |  |  |  | 11-17 | 3 (19) |  |  |  |  |  |  |  |  |  |  |  | |  |  |  |
|  |  |  |  | 12-16 | 2 (12) |  |  |  |  |  |  |  |  |  |  |  | |  |  |  |
|  |  |  |  | 12-17 | 1 (6) |  |  |  |  |  |  |  |  |  |  |  | |  |  |  |
|  |  |  |  |  |  |  |  |  |  |  |  |  |  |  |  |  | |  |  |  |
| **ISCOLE study** |  |  |  |  |  |  |  |  |  |  |  |  |  |  |  |  | |  |  |  |
| **WHO area** | **Number of countries with data** | **Survey/study used** | **n (%)** | **Age ranges** | **n (%)** | **Year of data collection** | |  | **Sample size** |  |  |  | **Prevalence of PA (%)** | |  |  | |  | **Male** | **Female** |
|  |  |  |  |  |  | **Average** | **Most recent** | **Least recent** | **Average** | **Median** | **Max** | **Min** | **Average** | **Max** | **Country with max** | **Min** | | **Country with Min** | **Average daily MVPA (min)** | **Average daily MVPA (min)** |
| **Central and eastern Europe** | 0 |  |  |  |  |  |  |  |  |  |  |  |  |  |  |  | |  |  |  |
| **Central Asia, Middle East, and North Africa** | 0 |  |  |  |  |  |  |  |  |  |  |  |  |  |  |  | |  |  |  |
| **East and Southeast Asia** | 1 | ISCOLE | - | 9-11 | 1 (100) | 2012 |  |  | 465 |  |  |  | 15.1 |  |  |  | |  | 50 | 41 |
| **High-income Asia Pacific** | 0 |  |  |  |  |  |  |  |  |  |  |  |  |  |  |  | |  |  |  |
| **High-income western countries** | 6 | ISCOLE | - | 9-11 | 6 (100) | 2012 | 2012 | 2012 | 529 | 458.5 | 639 | 414 | 45.3 | 61.4 | Finland | 26.5 | | USA | 70 | 53 |
| **Latin America and Caribbean** | 2 | ISCOLE | - | 9-11 | 2 (100) | 2012 | 2012 | 2012 | 646 | 645.5 | 822 | 469 | 51.7 | 59.5 | Colombia | 43.9 | | Brazil | 74 | 54 |
| **Oceania** | 0 |  |  |  |  |  |  |  |  |  |  |  |  |  |  |  | |  |  |  |
| **South Asia** | 1 | ISCOLE | - | 9-11 | 1 (100) | 2012 |  |  | 532 |  |  |  | 25.0 |  |  |  | |  | 62 | 38 |
| **Sub-Saharan Africa** | 3 | ISCOLE | - | 9-11 | 3 (100) | 2014 | 2017 | 2012 | 532 | 459 | 683 | 453 | 66.3 | 89 | Mozambique | 51.7 | | South Africa | 86.8 | 71 |
|  |  |  |  |  |  |  |  |  |  |  |  |  |  |  |  |  | |  |  | |
| **Global Matrix 3.0** |  |  |  |  |  |  |  |  |  |  |  |  |  |  |  |  | |  |  |  |
| **WHO area** | **Number of countries with data** | **Survey/study used** | **n (%)** | **Age ranges** | **n (%)** | **Year of data collection** | |  | **Sample size** |  |  |  | **Prevalence of PA (%)** | |  |  | |  | **Male** | **Female** |
|  |  |  |  |  |  | **Average** | **Most recent** | **Least recent** | **Average** | **Median** | **Max** | **Min** | **Regional average** | **Max** | **Country with max** | **Min** | | **Country with Min** | **Regional average (%)** | **Regional average (%)** |
| **Central and eastern Europe** | 6 | HBSC | 4 (36) | 7-18 | 1 (9) | 2014 | 2016 | 2011 | 5847 | 3802 | 38633 | 245 | 40%–46% | 80%–86% | Slovenia | 20%–26% | | Estonia & Poland | NA | NA |
|  |  | Regional study | 1 (9) | 9-17 | 1 (9) |  |  |  |  |  |  |  |  |  |  |  | |  |  |  |
|  |  | *Children’s Physical Activity Study 2015 and Schools in Motion Survey* | 1 (9) | 11-15 | 4 (36) |  |  |  |  |  |  |  |  |  |  |  | |  |  |  |
|  |  | *Lithuanian COSI study* | 1 (9) | 7-12 | 1 (9) |  |  |  |  |  |  |  |  |  |  |  | |  |  |  |
|  |  | Lifestyle study of school children made by Lithuanian Institute of Hygiene | 1 (9) | 10-17 | 1 (9) |  |  |  |  |  |  |  |  |  |  |  | |  |  |  |
|  |  | *ACDSi research project* | 1 (9) | 7-8 | 1 (9) |  |  |  |  |  |  |  |  |  |  |  | |  |  |  |
|  |  | Other national survey | 2 (18) | 10-17 | 1 (9) |  |  |  |  |  |  |  |  |  |  |  | |  |  |  |
|  |  |  |  | 5-19 | 1 (9) |  |  |  |  |  |  |  |  |  |  |  | |  |  |  |
| **Central Asia, Middle East, and North Africa** | 3 | GSHS | 2 (29) | 5-17 | 1 (14) | 2016 | 2017 | 2016 | 2990 | 1499 | 5862 | 183 | 20%–26% | 27%–33% | Lebanon & Qatar | <20% | | UAE | NA | NA |
|  |  | *Qatar National School Survey* | 1 (14) | 4-13 | 1 (14) |  |  |  |  |  |  |  |  |  |  |  | |  |  |  |
|  |  | *Lebanese-Food and Nutrition Security Study* | 1 (14) | 13-17 | 2 (29) |  |  |  |  |  |  |  |  |  |  |  | |  |  |  |
|  |  | Other national survey | 1 (14) | 6-12 | 2 (29) |  |  |  |  |  |  |  |  |  |  |  | |  |  |  |
|  |  | Local study | 2(29) | 13-17 | 1 (14) |  |  |  |  |  |  |  |  |  |  |  | |  |  |  |
| **East and Southeast Asia** | 4 | PAFCTYS | 1 (17) | 9-17 | 1 (17) | 2013 | 2016 | 2010 | 24481 | 2517 | 125281 | 263 | 20%–26% | 40%–46% | Hong-Kong | <20% | | China & Taiwan | NA | NA |
|  |  | *Nutrition and Health Survey* | 1 (17) | 7-15 | 1 (17) |  |  |  |  |  |  |  |  |  |  |  | |  |  |  |
|  |  | *National Physical Fitness Survey* | 1 (17) | 7-19 | 1 (17) |  |  |  |  |  |  |  |  |  |  |  | |  |  |  |
|  |  | IPEN | 1 (17) | 11-18 | 1 (17) |  |  |  |  |  |  |  |  |  |  |  | |  |  |  |
|  |  | *Thailand Physical Activity surveillance system* | 1 (17) | 6-8 | 1 (17) |  |  |  |  |  |  |  |  |  |  |  | |  |  |  |
|  |  | Local study | 1(17) | 6-17 | 1 (17) |  |  |  |  |  |  |  |  |  |  |  | |  |  |  |
| **High-income Asia Pacific** | 1 | *Korea Youth Risk Behavior web-based Survey* | 1 (100) | 12-17 | 1 (100) | 2017 |  |  | 57884 |  |  |  | <20% |  |  |  | |  | NA | NA |
| **High-income western countries** | 18 | HBSC | 7 (15) | 10-15 | 1 (2) | 2015 | 2018 | 2006 | 8928 | 1757 | 95591 | 91 | 27%–33% | 47%–53% | Netherlands | <20% | | Belgium & Scotland | |  |
|  |  | IDEFICS study | 2 (4) | 10-16 | 1 (2) |  |  |  |  |  |  |  |  |  |  |  | |  |  |  |
|  |  | CHAMPS study | 1 (2) | 10-17 | 2 (4) |  |  |  |  |  |  |  |  |  |  |  | |  |  |  |
|  |  | CHMS | 1 (2) | 11-12 | 1 (2) |  |  |  |  |  |  |  |  |  |  |  | |  |  |  |
|  |  | INCA3 | 1 (2) | 11-15 | 7 (15) |  |  |  |  |  |  |  |  |  |  |  | |  |  |  |
|  |  | ESTEBAN | 1 (2) | 12-16 | 1 (2) |  |  |  |  |  |  |  |  |  |  |  | |  |  |  |
|  |  | KiGGs Study | 1 (2) | 12-17 | 1 (2) |  |  |  |  |  |  |  |  |  |  |  | |  |  |  |
|  |  | ISCOLE | 1 (2) | 13-15 | 2 (4) |  |  |  |  |  |  |  |  |  |  |  | |  |  |  |
|  |  | YRBSSS | 1 (2) | 14-18 | 1 (2) |  |  |  |  |  |  |  |  |  |  |  | |  |  |  |
|  |  | NHANES | 1 (2) | 15-17 | 1 (2) |  |  |  |  |  |  |  |  |  |  |  | |  |  |  |
|  |  | NaSSDA | 1 (2) | 15.6 | 1 (2) |  |  |  |  |  |  |  |  |  |  |  | |  |  |  |
|  |  | Other national survey | 13 (28) | 16-17 | 2 (4) |  |  |  |  |  |  |  |  |  |  |  | |  |  |  |
|  |  | Regional study | 12 (26) | 2-11 | 1 (2) |  |  |  |  |  |  |  |  |  |  |  | |  |  |  |
|  |  | Local study | 4 (9) | 3-14 | 3 (6) |  |  |  |  |  |  |  |  |  |  |  | |  |  |  |
|  |  |  |  | 3-17 | 2 (4) |  |  |  |  |  |  |  |  |  |  |  | |  |  |  |
|  |  |  |  | 4-17 | 1 (2) |  |  |  |  |  |  |  |  |  |  |  | |  |  |  |
|  |  |  |  | 5-15 | 2 (4) |  |  |  |  |  |  |  |  |  |  |  | |  |  |  |
|  |  |  |  | 5-16 | 1 (2) |  |  |  |  |  |  |  |  |  |  |  | |  |  |  |
|  |  |  |  | 5-17 | 2 (4) |  |  |  |  |  |  |  |  |  |  |  | |  |  |  |
|  |  |  |  | 5-9 | 1 (2) |  |  |  |  |  |  |  |  |  |  |  | |  |  |  |
|  |  |  |  | 6-14 | 1 (2) |  |  |  |  |  |  |  |  |  |  |  | |  |  |  |
|  |  |  |  | 6-17 | 2 (4) |  |  |  |  |  |  |  |  |  |  |  | |  |  |  |
|  |  |  |  | 6-19 | 1 (2) |  |  |  |  |  |  |  |  |  |  |  | |  |  |  |
|  |  |  |  | 6-9 | 3 (6) |  |  |  |  |  |  |  |  |  |  |  | |  |  |  |
|  |  |  |  | 7-17 | 1 (2) |  |  |  |  |  |  |  |  |  |  |  | |  |  |  |
|  |  |  |  | 8-18 | 2 (4) |  |  |  |  |  |  |  |  |  |  |  | |  |  |  |
|  |  |  |  | 9-11 | 1 (2) |  |  |  |  |  |  |  |  |  |  |  | |  |  |  |
|  |  |  |  | 9-15 | 1 (2) |  |  |  |  |  |  |  |  |  |  |  | |  |  |  |
| **Latin America and Caribbean** | 7 | GSHS | 2 (25) | 13-17 | 1 (13) | 2015 | 2018 | 2012 | 40190 | 7217 | 256806 | 156 | 27%–33% | 34%–39% | Colombia | 20%–26% | | Chile | NA | NA |
|  |  | ENSIN | 1 (13) | 9-17 | 1 (13) |  |  |  |  |  |  |  |  |  |  |  | |  |  |  |
|  |  | ENSANUT | 1 (13) | 10-19 | 1 (13) |  |  |  |  |  |  |  |  |  |  |  | |  |  |  |
|  |  | *Latin American Study of Nutrition and Health* | 1 (13) | 11-18 | 1 (13) |  |  |  |  |  |  |  |  |  |  |  | |  |  |  |
|  |  | PeNSE | 1 (13) | 13-15 | 1 (13) |  |  |  |  |  |  |  |  |  |  |  | |  |  |  |
|  |  | Other national survey | 2 (25) | 6-17 | 1 (13) |  |  |  |  |  |  |  |  |  |  |  | |  |  |  |
|  |  |  |  | 10-17 | 1 (13) |  |  |  |  |  |  |  |  |  |  |  | |  |  |  |
|  |  |  |  | 15-19 | 1 (13) |  |  |  |  |  |  |  |  |  |  |  | |  |  |  |
| **Oceania** | 0 |  |  |  |  |  |  |  |  |  |  |  |  |  |  |  | |  | NA | NA |
| **South Asia** | 3 | GSHS | 3 (43) | 13-17 | 2 (28) | 2011 | 2015 | 2007 | 2660 | 1842 | 6529 | 241 | 34%–39% | 40%–46% | Bangladesh | 27%–33% | | India | NA | NA |
|  |  | ISCOLE | 1 (14) | 13-15 | 1 (14) |  |  |  |  |  |  |  |  |  |  |  | |  |  |  |
|  |  | *Chronic Disease Risk Factor study* | 1 (14) | 9-11 | 1 (14) |  |  |  |  |  |  |  |  |  |  |  | |  |  |  |
|  |  | Local study | 2 (28) | 12-18 | 1 (14) |  |  |  |  |  |  |  |  |  |  |  | |  |  |  |
|  |  |  |  | 15-19 | 1 (14) |  |  |  |  |  |  |  |  |  |  |  | |  |  |  |
|  |  |  |  | 5-14 | 1 (14) |  |  |  |  |  |  |  |  |  |  |  | |  |  |  |
| **Sub-Saharan Africa** | 6 | GSHS | 2 (20) | 13-15 | 2 (20) | 2014 | 2018 | 2005 | 2056 | 882 | 7348 | 239 | 47%–53% | 54%–59% | Zimbabwe | 27%–33% | | Ethiopia | NA | NA |
|  |  | ISCOLE | 1 (10) | 5-17 | 1 (10) |  |  |  |  |  |  |  |  |  |  |  | |  |  |  |
|  |  | *Disease, Activity and Schoolchildren’s Health study* | 1 (10) | 9-15 | 1 (10) |  |  |  |  |  |  |  |  |  |  |  | |  |  |  |
|  |  | *Mokola hypertension initiative* | 1 (10) | 3-17 | 1 (10) |  |  |  |  |  |  |  |  |  |  |  | |  |  |  |
|  |  | Other national survey | 1 (10) | 10-18 | 1 (10) |  |  |  |  |  |  |  |  |  |  |  | |  |  |  |
|  |  | Local study | 2 (20) | 13-14 | 1 (10) |  |  |  |  |  |  |  |  |  |  |  | |  |  |  |
|  |  | Expert opinion | 2 (20) | 9-11 | 1 (10) |  |  |  |  |  |  |  |  |  |  |  | |  |  |  |
|  |  |  |  | 8-14 | 1 (10) |  |  |  |  |  |  |  |  |  |  |  | |  |  |  |
|  |  |  |  | 8-12 | 1 (10) |  |  |  |  |  |  |  |  |  |  |  | |  |  |  |
|  |  |  |  |  |  |  |  |  |  |  |  |  |  |  |  |  | |  |  |  |
| **2017/2018 HBSC** |  |  |  |  |  |  |  |  |  |  |  |  |  |  |  |  | |  |  |  |
| **WHO area** | **Number of countries with data** | **Survey/study used** | **n (%)** | **Age ranges** | **n (%)** | **Year of data collection** | |  | **Sample size** |  |  |  | **Prevalence of PA (%)** | |  |  | |  |  |  |
|  |  |  |  |  |  | **Average** | **Most recent** | **Least recent** | **Average** | **Median** | **Max** | **Min** | **Regional average** | **Male regional average (%)** | **Male max % (country)** | **Male min % (country)** | | **Female regional average (%)** | **Female max % (country)** | **Female min % (country)** |
| **Central and eastern Europe** | 17 | HBSC | - | 11-15 | 17 (100) | 2018 |  |  | 1648 | 1551 | 3954 | 273 | NA | 24.8 | 42.7 (Serbia) | 16.7 (Republic of Moldova) | | 17.3 | 26.3 (Serbia) | 10.3 (Romania) |
| **Central Asia, Middle East, and North Africa** | 4 | HBSC | - | 11-15 | 4 (100) | 2018 |  |  | 1532 | 1556 | 1704 | 1345 | NA | 28.1 | 36.3 (Kazakhstan) | 20.7 (Azerbaijan) | | 21.7 | 32.3 (Kazakhstan) | 13.7 (Azerbaijan) |
| **East and Southeast Asia** | 0 |  |  |  |  |  |  |  |  |  |  |  | NA |  |  |  | |  |  |  |
| **High-income Asia Pacific** | 0 |  |  |  |  |  |  |  |  |  |  |  | NA |  |  |  | |  |  |  |
| **High-income western countries** | 25 | HBSC | - | 11-15 | 25 (100) | 2018 |  |  | 1797 | 1411 | 6193 | 314 | NA | 21.1 | 42.5 (Finland) | 12.5 (Denmark) | | 13.5 | 24.7 (Finland) | Italy (5.3) |
| **Latin America and Caribbean** | 0 |  |  |  |  |  |  |  |  |  |  |  | NA |  |  |  | |  |  |  |
| **Oceania** | 0 |  |  |  |  |  |  |  |  |  |  |  | NA |  |  |  | |  |  |  |
| **South Asia** | 0 |  |  |  |  |  |  |  |  |  |  |  | NA |  |  |  | |  |  |  |
| **Sub-Saharan Africa** | 0 |  |  |  |  |  |  |  |  |  |  |  | NA |  |  |  | |  |  |  |
|  |  |  |  |  |  |  |  |  |  |  |  |  |  |  |  |  | |  |  |  |
| **GSHS WHO Fact Sheets & Reports** |  |  |  |  |  |  |  |  |  |  |  |  |  |  |  |  | |  |  |  |
| **WHO area** | **Number of countries with data** | **Survey/study used** | **n (%)** | **Age ranges** | **n (%)** | **Year of data collection** | |  | **Sample size** |  |  |  | **Prevalence of PA (%)** | |  |  | |  |  |  |
|  |  |  |  |  |  | **Average** | **Most recent** | **Least recent** | **Average** | **Median** | **Max** | **Min** | **Regional average** | **Max** | **Country with max** | **Min** | | **Country with Min** | **Male regional average (%)** | **Female regional average (%)** |
| **Central and eastern Europe** | 1 | GSHS | - | 13-16+ | 1 (100) | 2007 |  |  | 1409 |  |  |  | 14.1 |  |  |  | |  | 17.2 | 13.0 |
| **Central Asia, Middle East, and North Africa** | 17 | GSHS | - | 13-17 | 8 (47) | 2012 | 2017 | 2007 | 3660 | 2870 | 7141 | 1908 | 16.7 | 37.6 | Mongolia | 9.2 | | Lebanon | 21.1 | 12.3 |
|  |  |  |  | 13-15 | 9 (53) |  |  |  |  |  |  |  |  |  |  |  | |  |  |  |
| **East and Southeast Asia** | 14 | GSHS | - | 13-17 | 14 (82) | 2013 | 2016 | 2003 | 6568 | 3755 | 25507 | 2599 | 14.4 | 28.7 | Taiwan | 7.3 | | Philippines | 18.9 | 9.9 |
|  |  |  |  | 13-15 | 3 (18) |  |  |  |  |  |  |  |  |  |  |  | |  |  |  |
| **High-income Asia Pacific** | 0 |  |  |  |  |  |  |  |  |  |  |  |  |  |  |  | |  |  |  |
| **High-income western countries** | 0 |  |  |  |  |  |  |  |  |  |  |  |  |  |  |  | |  |  |  |
| **Latin America and Caribbean** | 32 | GSHS | - | 13-17 | 9 (28) | 2010 | 2017 | 2003 | 2727 | 2037 | 28368 | 212 | 18.0 | 31.8 | Antigua and Barbuda | 8.1 | | Venezuela | 21.6 | 14.5 |
|  |  |  |  | 13-15 | 23 (72) |  |  |  |  |  |  |  |  |  |  |  | |  |  |  |
| **Oceania** | 13 | GSHS | - | 13-17 | 7 (54) | 2014 | 2017 | 2010 | 1650 | 1421 | 3705 | 140 | 18.3 | 31.5 | Niue | 10.6 | | Tuvalu | 21.1 | 15.2 |
|  |  |  |  | 13-15 | 6 (46) |  |  |  |  |  |  |  |  |  |  |  | |  |  |  |
| **South Asia** | 6 | GSHS | - | 13-17 | 4 (67) | 2013 | 2016 | 2007 | 5499 | 5860.5 | 8130 | 2579 | 20.7 | 43.0 | Bangladesh | 8.8 | | Afghanistan | 21.9 | 17.8 |
|  |  |  |  | 13-15 | 2 (33) |  |  |  |  |  |  |  |  |  |  |  | |  |  |  |
| **Sub-Saharan Africa** | 17 | GSHS | - | 13-17 | 7 (41) | 2009 | 2017 | 2003 | 2547 | 2234 | 4531 | 1648 | 16.8 | 33.4 | Ghana | 9.2 | | Liberia | 19.2 | 13.7 |
|  |  |  |  | 13-15 | 9 (53) |  |  |  |  |  |  |  |  |  |  |  | |  |  |  |
|  |  |  |  | <12-18+ | 1 (6) |  |  |  |  |  |  |  |  |  |  |  | |  |  |  |
|  |  |  |  |  |  |  |  |  |  |  |  |  |  |  |  |  | |  |  |  |
| **Xu et al. (2020)** |  |  |  |  |  |  |  |  |  |  |  |  |  |  |  |  | |  |  |  |
| **WHO area** | **Number of countries with data** | **Survey/study used** | **n (%)** | **Age ranges** | **n (%)** | **Year of data collection** | |  | **Sample size** |  |  |  | **Prevalence of PA (%)** | |  |  | |  | **Male** | **Female** |
|  |  |  |  |  |  | **Average** | **Most recent** | **Least recent** | **Average** | **Median** | **Max** | **Min** | **Regional average** | **Max** | **Country with max** | **Min** | | **Country with Min** | **Regional average (%)** | **Regional average (%)** |
| **Central and eastern Europe** | 0 |  |  |  |  |  |  |  |  |  |  |  |  |  |  |  | |  |  |  |
| **Central Asia, Middle East, and North Africa** | 10 | GSHS | - | 12-15 | 10 (100) | 2012 | 2015 | 2010 | 2295 | 2189.5 | 3720 | 1553 | 15.86 | 26.9 | Mongolia | 10.5 | | Qatar | 20.1 | 12.0 |
| **East and Southeast Asia** | 9 | GSHS | - | 12-15 | 9 (100) | 2014 | 2015 | 2012 | 4910 | 1827 | 16287 | 1664 | 11.2 | 16.2 | Laos | 6.3 | | Cambodia | 15.1 | 7.5 |
| **High-income Asia Pacific** | 0 |  |  |  |  |  |  |  |  |  |  |  |  |  |  |  | |  |  |  |
| **High-income western countries** | 0 |  |  |  |  |  |  |  |  |  |  |  |  |  |  |  | |  |  |  |
| **Latin America and Caribbean** | 17 | GSHS | - | 12-15 | 17(100) | 2011 | 2015 | 2009 | 3121 | 1644 | 21762 | 1062 | 16.3 | 22.4 | Antigua and Barbuda | 11.1 | | Guatemala | 20.7 | 12.3 |
| **Oceania** | 6 | GSHS | - | 12-15 | 6 (100) | 2012 | 2015 | 2010 | 1350 | 1168.5 | 2213 | 725 | 14.1 | 17.1 | Kiribati | 10.7 | | Vanuatu | 14.7 | 13.6 |
| **South Asia** | 3 | GSHS | - | 12-15 | 6 (100) | 2012 | 2014 | 2009 | 3098 | 2760 | 5005 | 1530 | 20.9 | 42.4 | Bangladesh | 8.8 | | Afghanistan | 20.9 | 20.8 |
| **Sub-Saharan Africa** | 8 | GSHS | - | 12-15 | 8 (100) | 2012 | 2015 | 2009 | 1680 | 1729 | 2643 | 697 | 15.6 | 24.4 | Benin | 7.8 | | Sudan | 18.2 | 12.8 |
|  |  |  |  |  |  |  |  |  |  |  |  |  |  |  |  |  | |  |  |  |
| **ICAD** |  |  |  |  |  |  |  |  |  |  |  |  |  |  |  |  | |  |  |  |
| **WHO area** | **Number of countries with data** | **Survey/study used** | **n (%)** | **Age ranges** | **n (%)** | **Year of data collection** | |  | **Sample size** |  |  |  | **Prevalence of PA (%)** | |  |  | |  | **Male** | **Female** |
|  |  |  |  |  |  | **Average** | **Most recent** | **Least recent** | **Average** | **Median** | **Max** | **Min** | **Regional average** | **Max** | **Country with max** | **Min** | | **Country with Min** | **Regional average (%)** | **Regional average (%)** |
| **Central and eastern Europe** | 1 | ICAD analysis | - | 9-10 | 1 (100) | 1998-1999 |  |  | 643 |  |  |  |  |  |  |  | |  | 26 | 18 |
| **Central Asia, Middle East, and North Africa** | 0 |  |  |  |  |  |  |  |  |  |  |  |  |  |  |  | |  |  |  |
| **East and Southeast Asia** | 0 |  |  |  |  |  |  |  |  |  |  |  |  |  |  |  | |  |  |  |
| **High-income Asia Pacific** | 0 |  |  |  |  |  |  |  |  |  |  |  |  |  |  |  | |  |  |  |
| **High-income western countries** | 6 | ICAD analysis | - | 9-10 | 6 (67) | N/A | 2006-2009 | 1999-2000 | 1421 | 620.5 | 6407 | 127 |  |  |  |  | |  | 12.1 | 4.1 |
|  |  |  |  | 12-13 | 3 (33) |  |  |  |  |  |  |  |  |  |  |  | |  |  |  |
| **Latin America and Caribbean** | 1 | ICAD analysis | - | 12-13 | 1 (100) | 2006-2007 |  |  | 420 |  |  |  |  |  |  |  | |  | 7 | 2 |
| **Oceania** | 0 |  |  |  |  |  |  |  |  |  |  |  |  |  |  |  | |  |  |  |
| **South Asia** | 0 |  |  |  |  |  |  |  |  |  |  |  |  |  |  |  | |  |  |  |
| **Sub-Saharan Africa** | 0 |  |  |  |  |  |  |  |  |  |  |  |  |  |  |  | |  |  |  |
|  |  |  |  |  |  |  |  |  |  |  |  |  |  |  |  |  | |  |  |  |
| **Marques et al. (2020)** |  |  |  |  |  |  |  |  |  |  |  |  |  |  |  |  | |  |  |  |
| **WHO area** | **Number of countries with data** | **Survey/study used** | **n (%)** | **Age ranges** |  | **Year of data collection** | |  | **Sample size** |  |  |  | **Prevalence of PA (%)** | |  |  | |  | **Male** | **Female** |
|  |  |  |  | **Ranges** | **n (%)** | **Average** | **Most recent** | **Least recent** | **Average** | **Median** | **Max** | **Min** | **Regional average** | **Max** | **Country with max** | **Min** | | **Country with Min** | **Regional average (%)** | **Regional average (%)** |
| **Central and eastern Europe** | 14 | NA |  | 11-17 | 14 (100) | NA |  |  | NA |  |  |  | 22.9 | 28.6 | Bulgaria | 16.4 | | Estonia | 28.6 | 18.5 |
| **Central Asia, Middle East, and North AFrica** | 13 | NA |  | 11-17 | 13 (100) | NA |  |  | NA |  |  |  | 16.4 | 25.2 | Mongolia | 10.6 | | Syria | 21.7 | 12.5 |
| **East and Southeast Asia** | 8 | NA |  | 11-17 | 8 (100) | NA |  |  | NA |  |  |  | 11.4 | 15.2 | Laos | 7.3 | | Cambodia | 17.8 | 7.9 |
| **High-income Asia Pacific** | 0 |  |  |  |  |  |  |  |  |  |  |  |  |  |  |  | |  |  |  |
| **High-income western countries** | 25 | NA |  | 11-17 | 25 (100) | NA |  |  | NA |  |  |  | 18.5 | 32.3 | USA | 10.3 | | Italy | 24.7 | 14.4 |
| **Latin America and Caribbean** | 22 | NA |  | 11-17 | 22 (100) | NA |  |  | NA |  |  |  | 16.5 | 22.7 | Antigua and Barbuda | 11.7 | | Curaçao | 19.1 | 9.4 |
| **Oceania** | 11 | NA |  | 11-17 | 11 (100) | NA |  |  | NA |  |  |  | 14.8 | 24 | Tokelau | 11 | | Vanuatu | 15.7 | 13.4 |
| **South Asia** | 3 | NA |  | 11-17 | 3 (100) | NA |  |  | NA |  |  |  | 23.3 | 48.2 | Bangladesh | 10.1 | | Afghanistan | 20.2 | 24 |
| **Sub-Saharan Africa** | 8 | NA |  | 11-17 | 8 (100) | NA |  |  | NA |  |  |  | 14.7 | 20.2 | Tanzania | 8.8 | | Sudan | 18.3 | 12.5 |

Notes: PA = Physical activity; Max = maximum; Min = minimum; HBSC = Health Behaviour in School-aged Children; GSHS = Global School Health Survey; KYRBS = Korea Youth Risk Behavior Web-based Survey; NNPAS = National Nutrition and Physical Activity Survey; YRBSS = Youth Risk Behavior Surveillance System; PeNSE = National Adolescent School-based Health Survey in Brazil; ENSANUT = Encuesta Nacional de Salud y Nutrición; ISCOLE = International Study of Childhood Obesity, Lifestyle and the Environment; COSI = WHO European Childhood Obesity Surveillance Initiative; ACDSi = analysis of Children’s Development in Slovenia; PAFCTYS = Physical Activity and Fitness in China—The Youth Study; IPEN = International Physical Activity and the Environment; NaSSDA = National Secondary Students' Diet and Activity; CHAMPS = Child Health and Mortality Prevention Surveillance; CHMS = Canadian Health Measures Survey; LITU = ; INCA3 = Étude Individuelle Nationale des Consommations Alimentaires 3; ESTEBAN = Étude de Santé sur l’Environnement, la Biosurveillance, l’Activité Physique et la Nutrition; KiGGs = German Health Interview and Examination Survey for Children and Adolescents; GINI+ = ; IDEFICS = Identification and prevention of Dietary- and lifestyle-induced health EFfects In Children and infantS; NHANES = National Health and Nutrition Examination Survey; ENSIN = Encuesta Nacional de Situación Nutricional; NA = information not available; WHO World Health Organisation.
