# Supplementary material for: Global prevalence of physical activity for children and adolescents; inconsistencies, research gaps, and recommendations: a narrative review
Source: Int J Behav Nutr Phys Act. 2021 Jun 29;18:81. doi: 10.1186/s12966-021-01155-2 (PMC8243483; doi:10.1186/s12966-021-01155-2)
Supplement: Supplementary file 4 — Additional file 4. Extraction of physical activity questionnaire items, translation, validity, and reliability information for the GSHS and the HBSC surveys [file 12966_2021_1155_MOESM4_ESM.docx]

**Online Supplement File 4: Extraction of physical activity questionnaire items, translation, validity, and reliability information for the GSHS and the HBSC surveys**

**GSHS**

A variety of information concerning the background and methods of the GSHS survey can be found on the World Health Organisation (WHO) website and on the Centers for Disease Control and Prevention (CDC) website as the GSHS survey was developed by the WHO and CDC in collaboration with UNICEF, UNESCO, and UNAIDS.^1^

**Item(s) measuring frequency/intensity/dose of any physical activity domain**

GHSH Core questionnaire

“*The next 3 questions ask about physical activity. Physical activity is any activity that increases your heart rate and makes you breathe hard. Physical activity can be done in sports, playing with friends, or walking to school. Some examples of physical activity are running, fast walking, biking, dancing, football, and COUNTRY SPECIFIC EXAMPLES.*

1. *During the past 7 days, on how many days were you physically active for a total of at least 60 minutes per day? ADD UP ALL THE TIME YOU SPENT IN ANY KIND OF PHYSICAL ACTIVITY EACH DAY*

*A) 0 days*

*B) 1 day*

*C) 2 days*

*D) 3 days*

*E) 4 days*

*F) 5 days*

*G) 6 days*

*H) 7 days*

*Core-expanded questions*

1. *During the past 7 days, on how many days did you do exercises to strengthen or tone your muscles, such as push-ups, sit-ups, or weightlifting?*

*A) 0 days*

*B) 1 day*

*C) 2 days*

*D) 3 days*

*E) 4 days*

*F) 5 days*

*G) 6 days*

*H) 7 days*

1. *During the past 7 days, on how many days did you do stretching exercises, such as toe touching, knee bending, or leg stretching?*

*A) 0 days*

*B) 1 day*

*C) 2 days*

*D) 3 days*

*E) 4 days*

*F) 5 days*

*G) 6 days*

*H) 7 days*”^2^

The first physical activity item is similar to the first physical activity item of the 2019 Youth Risk Behavior Surveillance System (YRBSS) questionnaire, a survey that was also developed by CDC.

**Official target population**

GSHS is a school-based survey conducted primarily among students aged 13–17 years-old. Answers are self-reported.^3^

**Languages available**

English and Spanish versions are the official versions of the GSHS questionnaire.^2^ Datasets are available for 101 countries in Africa, Americas, Southeast Asia, Europe, Mediterranean region and Western Pacific.^4^ The following version of the GSHS questionnaire are available on the WHO website:

English: <https://www.who.int/ncds/surveillance/gshs/GSHS_Core_Modules_2013_English.pdf>

Spanish: <https://www.who.int/ncds/surveillance/gshs/GSHS_Core_Modules_2013_Spanish.pdf>

Arabic: <https://www.who.int/ncds/surveillance/gshs/2010_Algeria_GSHS_Questionnaire_AR.pdf>

French: <https://www.who.int/ncds/surveillance/gshs/2016_GSHS_Benin_Questionnaire_FR.pdf>

Kiswahili: <https://www.who.int/ncds/surveillance/gshs/2014_GSHS_Tanzania_Questionnaire_Kiswahili.pdf>

Creole : <https://www.who.int/ncds/surveillance/gshs/2014_Haiti_GSHS_qs_Creole.pdf>

Dari: <https://www.who.int/ncds/surveillance/gshs/2014_Afghanistan_GSHS_Questionnaire_Dari.pdf>

Albanian: <https://www.who.int/ncds/surveillance/gshs/2007_Macedonia_GSHS_Questionnaire_Albanian.pdf>

Macedonian: <https://www.who.int/ncds/surveillance/gshs/2007_Macedonia_GSHS_Questionnaire_Macedonian.pdf>

Russian: <https://www.who.int/ncds/surveillance/gshs/2005%20Russian%20Tajikistan%20GSHS%20questionnaire.pdf>

Hindi: <https://www.who.int/ncds/surveillance/gshs/2007_India_GSHS_Questionnaire_Hindi.pdf>

Nepali: <https://www.who.int/ncds/surveillance/gshs/2015_GSHS_Nepal_Questionnaire-Nepali.pdf>

Tetum: <https://www.who.int/ncds/surveillance/gshs/2014-GSHS-Timor-Leste-Questionnaire-Tetum.pdf>

Malay: <https://www.who.int/ncds/surveillance/gshs/2014_GSHS_Questionnaire_Brunei_Darussalam_Malay.pdf>

Khmer: <https://www.who.int/ncds/surveillance/gshs/Cambodia_2013_GSHS_Questionnaire_Khmer.pdf>

Chinese: <https://www.who.int/ncds/surveillance/gshs/China%20questionnaire%20chinese%202003.pdf>

Vietnamese: <https://www.who.int/ncds/surveillance/gshs/2013_Vietnam_GSHS_Questionnaire_Vietnamese.pdf>

Portuguese: [2013_Mozambique_GSHS_Questionnaire_Portuguese.pdf](https://extranet.who.int/ncdsmicrodata/index.php/catalog/547/download/4085)

Niue: [2009_Niue_Language_GSHS_Questionnaire.pdf](https://extranet.who.int/ncdsmicrodata/index.php/catalog/201/download/1585)

Sinhala: [2016_Sri_Lanka_GSHS_Questionnaire (Sinhala).pdf](https://extranet.who.int/ncdsmicrodata/index.php/catalog/648/download/4644)

World Health Organization. Global school-based student health survey (GSHS). WHO CHP. 2009. <https://www.who.int/ncds/surveillance/gshs/en/>

**Translation/ cultural adaptation methods info**

No official GSHS or WHO information was found concerning the methods for the translation/cultural adaptation of the questionnaire used in the GSHS survey. However, the following information was found concerning the translation methods for the English to Fijian version: “*The selected GSHS items were translated from English into the local vernacular Fijian language by a bilingual native speaker, pilot-tested with 21 adolescent and adult ethnic Fijian respondents, modified and edited for clarity, and then back-translated into English by a bilingual scholar of Fijian languages. The back-translated and original versions were compared and the Fijian version was then edited to achieve consistency across the two versions. The penultimate Fijian language translation was reviewed and edited by a native speaker for grammatical and idiomatic accuracy and resulted in the final version*”.^5^

A similar method was reported for the English to Persian translation of the GSHS survey questionnaire: “*Both GSHS and GYTS plus Shisha Modules questionnaires were translated from English into the Persian language by a native English-Persian speaker, and then tested with 10 adolescents as a pilot, edited and reformed for clarity, and then back-translated into English by a bilingual researcher of Persian language. The original and back-translated questionnaires were compared and the Persian version was then corrected to reach consistency in both versions. The translated Persian version was checked again by a native speaker for colloquial and grammatical correctness, resulting in the final questionnaire*.”^6^

**Validity and Reliability study**

According to the CDC (2013),^7^ no study has been conducted to assess the validity of all self-reported behaviors that are included on the YRBSS questionnaire. A literature review assessing cognitive and situational factors that might affect the validity of adolescent self-reporting of behaviors measured by the YRBSS questionnaire was conducted by CDC in 2003.^8^ CDC concluded that “*although* *self-reports of these types of behaviors are affected by both cognitive and situational factors, these factors do not threaten the validity of self-reports of each type of behavior equally*. *In addition, each type of behavior differs in the extent to which its self-report can be validated by an objective measure. […] Understanding the differences in factors that compromise the validity of self-reporting of different types of behavior can assist policymakers in interpreting data and researchers in designing measures that do not compromise validity”.*^7^

A study evaluated the test-retest reliability with kappa coefficients (k), % agreement, and prevalence estimates of the Fiji translated and adapted version of the GSHS survey questionnaire. Authors found a moderate agreement with a Kappa coefficient of 0.42 and 56% agreement.^5^ Another study assessed the reliability of the Persian version of GSHS survey adapted for Iranian school students in 2014 and found an acceptable reliability for the physical activity item (Kendall’s tau-b = 0.73).^6^

Note: The first physical activity item of the GHSH Core questionnaire (#1 presented above) is the same as the HBSC questionnaire “MVPA” item presented below. Hence, the reliability and validity information presented in the next part applies to that specific question for GSHS as well.

**HBSC**

**Item(s) measuring frequency/intensity/dose of any physical activity domain**

*All the information presented here was extracted from the Health Behaviour in School-Aged Children (HBSC) Study Protocol Background, Methodology and Mandatory Items for the 2017/18 Survey^9^

**“*Item box 1. MVPA***

*Physical activity is any activity that increases your heart rate and makes you get out of breath some of the time. Physical activity can be done in sports, school activities, playing with friends, or walking to school. Some examples of physical activity are running, brisk walking, rollerblading, biking, dancing, skateboarding, swimming, soccer, basketball, football and surfing [country-specific examples can be given].*

***Over the past 7 days, on how many days were you physically active for a total of at least 60 minutes per day?***

*Please* ***add up*** *all the time you spent in physical activity each day.*

*◯ 0 day*

*◯ 1 day*

*◯ 2 days*

*◯ 3 days*

*◯ 4 days*

*◯ 5 days*

*◯ 6 days*

*◯ 7 days*

***Item box 2. VPA***

*Outside school hours: how* ***often*** *do you usually exercise in your free time so much that you get out of breath or sweat?*

*Every day*

*◯ 4 to 6 times a week*

*◯ 2 to 3 times a week*

*◯ Once a week*

*◯ Once a month*

*◯ Less than once a month*

*◯ Never*”

**Official target population**

HBSC is a school-based survey conducted among students aged 11,13 and 15 years-old. Answers are self-reported.^9^

**Languages available**

We could not find an official list of the HBSC questionnaire available language, but the 2018 HBSC survey collected data in 45 countries located in Europe, Middle East, and North America.^10^

**Translation/ cultural adaptation methods information**

*All the information presented here was extracted from the Health Behaviour in School-Aged Children (HBSC) Study Protocol Background, Methodology and Mandatory Items for the 2017/18 Survey^9^ (p.16)

“*The source language for all items is English, with translations into national language(s). Accurate translation is crucial for robust cross-national comparison of survey results. The standard approach in HBSC has been to ask the same question in each country through direct translation, with adaptations permitted only when absolutely necessary for linguistic clarity. The standard method employed in the study for checking translations is a process in which the translated questions are back-translated into the source language (English) and compared against the original. This method (without additional reviewing techniques) has limitations, but it identifies major errors and highlights potential discrepancies. The back-translation process has been strengthened in recent surveys by incorporating a more thorough system through which back-translations are also independently checked by a translation team specifically established for the task, followed by discussion and further review involving the researcher and*

*translator where necessary.*

*New member countries carrying out the survey for the first time are required to test their translations through pilot surveys and qualitative work (such as focus groups with children). Translations are adjusted at this stage and may be further refined during the required pilot phase prior to each survey. New items are also thoroughly tested across countries in this way. Language groups have been in place from the early phase of the study, reflecting geographic zones used in the management structure: countries that include Russian-speaking populations, for example, have collaborated to work more efficiently and ensure consistency with translations*”.

**MVPA item validity and reliability**

*All the information presented here was extracted from the Health Behaviour in School-Aged Children (HBSC) Study Protocol Background, Methodology and Mandatory Items for the 2017/18 Survey^9^

The MVPA item was validated against seven-day continuous measurement using an accelerometer (r = 0.40, p<.001) and observed a substantial test-retest stability (intraclass correlation coefficient (ICC) = 0.77).^11^ Another study found that this self-reported MVPA index had an acceptable validity for measuring non-compliance with physical activity recommendations in 15–17-year-old adolescents.^12^ The specificity for meeting current MVPA guidelines assessed by the MVPA index ranged from 60.8% (for boys) to 79.7% (girls) when comparing five days of valid accelerometer wearing time.^12^

In addition, Acceptable test-retest stability was found to be in the samples of Finnish (ICC = 0.6-0.8),^13^ Chinese (ICC = 0.82),^14^ and Czech, Slovak and Polish (ICC = 0.6)^15^ 11–15-year-olds.

**VPA item validity and reliability**

*All the information presented here was extracted from the Health Behaviour in School-Aged Children (HBSC) Study Protocol Background, Methodology and Mandatory Items for the 2017/18 Survey^9^

A study showed that 13- and 15-year-old adolescents classified as active had higher fitness levels than those classified as inactive and reliability of the measure was good (67% to 85%).^16^ Another study found the VPA item to be reliable (ICC = 0.71) and classified its validity as fair when correlated with maximal oxygen consumption (r = 0.33).^17^ The test-retest reliability was assessed in China^14^ and in Czechia, Slovakia and Poland,^15^ resulting in the ICCs 0.68 and 0.62, respectively. A review indicated that the VPA item has strong reliability and validity but highlighted that validity against objective monitoring of PA is lacking as only fitness-related criterion measures have been used.^18^

**References**

1. Centers for Disease Control and Prevention. Global School-based Student Health Survey. https://www.cdc.gov/gshs/background/index.htm. Published 2016. Accessed May 28, 2019.

2. Questionnaire - CDC Global School-based Student Health Survey (GSHS). https://www.cdc.gov/gshs/questionnaire/index.htm. Accessed August 10, 2020.

3. CDC. *Global School-Based Student Health Survey Background*. www.cdc.gov/gshs. Accessed August 10, 2020.

4. NCDs | Global school-based student health survey (GSHS). *WHO*. 2020. http://www.who.int/ncds/surveillance/gshs/datasets/en/. Accessed August 7, 2020.

5. Becker AE, Roberts AL, Perloe A, et al. Youth health-risk behavior assessment in Fiji: the reliability of Global School-based Student Health Survey content adapted for ethnic Fijian girls. *Ethn Health*. 2010;15(2):181-197. doi:10.1080/13557851003615552

6. Ziaei R, Dastgiri S, Soares J, et al. Reliability and Validity of the Persian Version of Global School-based Student Health Survey Adapted for Iranian School Students. *J Clin Res governanace*. 2014;3(2):134-140. doi:10.13183/jcrg.v3i2.138

7. Centers for Disease Control and Prevention. *Methodology of the Youth Risk Behavior Surveillance System--2013.* Washington; 2013.

8. Brener ND, Billy JOG, Grady WR. Assessment of factors affecting the validity of self-reported health-risk behavior among adolescents: Evidence from the scientific literature. *J Adolesc Heal*. 2003;33(6):436-457. doi:10.1016/S1054-139X(03)00052-1

9. Currie C, Inchley J, Molcho M, Lenzi M, Veselska Z, Wild F. Health Behaviour in School-aged Children (HBSC) study protocol: Background, methodology and mandatory items for the 2013/14 survey. 2014. https://researchonline.gcu.ac.uk/en/publications/health-behaviour-in-school-aged-children-hbsc-study-protocol-back. Accessed August 7, 2020.

10. World Health Organization. *Spotlight on Adolescent Health and Well-Being. Findings from the 2017/2018 Health Behaviour in School-Aged Children (‎‎HBSC)‎‎ Survey in Europe and Canada. International Report. Volume 2. Key Data.* Geneva; 2020. https://apps.who.int/iris/handle/10665/332104.

11. Prochaska JJ, Sallis JF, Long B. A physical activity screening measure for use with adolescents in primary care. *Arch Pediatr Adolesc Med*. 2001;155(5):554-559. doi:10.1001/archpedi.155.5.554

12. Ridgers ND, Timperio A, Crawford D, Salmon J. Validity of a brief self-report instrument for assessing compliance with physical activity guidelines amongst adolescents. *J Sci Med Sport*. 2012;15(2):136-141. doi:10.1016/j.jsams.2011.09.003

13. Vuori M, Ojala K, Tynjälä J, Villberg J, Välimaa R, Kannas L. Liikunta-aktiivisuutta koskevien kysymysten stabiliteetti WHO-Koululaistutkimuksessa. *Liik T*. 2005;42(6):39-46. https://converis.jyu.fi/converis/portal/detail/Publication/15551646?auxfun=&lang=en_GB. Accessed August 10, 2020.

14. Liu Y, Wang M, Tynjälä J, et al. Test-retest reliability of selected items of health behaviour in school-aged children (HBSC) survey questionnaire in Beijing, China. *BMC Med Res Methodol*. 2010;10(1):1-9. doi:10.1186/1471-2288-10-73

15. Bobakova D, Hamrik Z, Badura P, Sigmundova D, Nalecz H, Kalman M. Test–retest reliability of selected physical activity and sedentary behaviour HBSC items in the Czech Republic, Slovakia and Poland. *Int J Public Health*. 2014;60(1):59-67. doi:10.1007/s00038-014-0628-9

16. Booth ML, Okely AD, Chey T, Bauman A. The reliability and validity of the physical activity questions in the WHO health behaviour in schoolchildren (HBSC) survey: A population study. *Br J Sports Med*. 2001;35(4):263-267. doi:10.1136/bjsm.35.4.263

17. Rangul V, Holmen TL, Kurtze N, Cuypers K, Midthjell K. Reliability and validity of two frequently used self-administered physical activity questionnaires in adolescents. *BMC Med Res Methodol*. 2008;8(1):1-10. doi:10.1186/1471-2288-8-47

18. Biddle SJH, Gorely T, Pearson N, Bull FC. An assessment of self-reported physical activity instruments in young people for population surveillance: Project ALPHA. *Int J Behav Nutr Phys Act*. 2011;8(1):1-9. doi:10.1186/1479-5868-8-1
